# Sex-specific effects of automated feeding on lifespan and reproduction in *Nothobranchius furzeri*

**DOI:** 10.3389/fragi.2026.1869084

**Published:** 2026-09-14

**Authors:** Luca Dolfi, Michael Kothmayer, Elmar Erwin Ebner, Renate Löhnert, Oliver Pusch

**Affiliations:** 1 Center for Anatomy and Cell Biology, Medical University of Vienna, Vienna, Austria; 2 BIO@SNS Laboratory, Scuola Normale Superiore of Pisa, Pisa, Italy; 3 Department of Biological Sciences and Pathobiology, Institute for Infectiology and Virology, University of Veterinary Medicine Vienna, Vienna, Austria

**Keywords:** African turquoise killifish, automated feeding system, lifespan, *Nothobranchius furzeri*, pelleted diet, sex specificity

## Abstract

**Introduction:**

*Nothobranchius furzeri*, commonly known as African turquoise killifish, has emerged as a powerful model organism in diverse biological research areas owing to its exceptionally short lifespan of 4‐6 months. Dietary choices can significantly impact the physiology of this fish and its median longevity, prompting considerations on live versus dry food and feeding frequencies.

**Methods:**

Here, we present a novel, cost‐effective, and modular automated feeding system for *N. furzeri*, capable of delivering up to 36 feedings per day with high precision. Using this system, we conducted a comprehensive, multi‐parametric analysis comparing the effects of three commercially available dry diets varying in protein and fat content and live chironomid larvae (bloodworms), administered either via high‐frequency automated feeding or manual twice‐daily feeding.

**Results:**

A continuous diet of 24‐36 feedings per day increased fish growth in weight but not in length compared to a standard twice‐daily regimen. Unexpectedly, while male lifespan remained unaffected by feeding regimen or diet, females subjected to high‐frequency automated feeding showed reduced longevity, particularly with high‐calorie diets. Additionally, pooled reproductive profiles suggested that reproductive output was associated with dietary fat and protein content as well as feeding frequency. Greater delivered food availability, particularly under high‐frequency feeding with energy‐rich diets, was associated with increased egg production, reduced embryo survival, and a shortened reproductive period.

**Discussion:**

Together, these findings provide important insights for optimizing husbandry and experimental design in *N. furzeri,* supporting its broader application as a high‐throughput vertebrate model.

## 1 Introduction

*Nothobranchius furzeri*, commonly known as the African turquoise killifish, has gained prominence over the past decade as a powerful model organism, serving as a focal point for diverse research studies across various fields of biology (Genade et al., 2005). These investigations span a wide array of topics, encompassing aging (Terzibasi et al., 2008; Valenzano et al., 2015), development and diapause (Dolfi et al., 2014; Dolfi et al., 2019; Podrabsky et al., 2010), epigenetics (Steiger et al., 2026; Zupkovitz et al., 2021; Zupkovitz et al., 2018), reproduction (Dolfi et al., 2021; Api et al., 2018), neurobiology and behavior (Tozzini et al., 2012). A central factor contributing to the success of *N. furzeri* as a model organism is its remarkably short lifespan of 4–6 months (Kim et al., 2016). This relatively short lifespan accelerates experimental timelines and the efficient acquisition of aging data, all within a vertebrate genetic framework (Harel et al., 2015).

Diet is an essential factor modulating lifespan, and studies on the relationship between diet and longevity have provided important insights into the mechanisms linking nutrition and aging (Hwangbo et al., 2020). Research has consistently demonstrated that dietary choices and regimens, such as caloric restriction (Xiang and He, 2011) or intermittent fasting (Longo et al., 2021), can profoundly impact longevity (Duregon et al., 2021; Anton and Leeuwenburgh, 2013).

The dietary preferences and requirements of *N. furzeri* constitute integral aspects of its biology. As an omnivorous fish species, *N. furzeri* feeding strategies encompass diverse diet niches in their natural habitats, including aquatic invertebrates and plant matter (Terzibasi Tozzini and Cellerino, 2020; Polačik and Reichard, 2010). Researchers studying this species in laboratory captivity face therefore choices regarding the composition of the fish’s diet, with these decisions significantly influencing experimental outcomes (Dodzian et al., 2018). A fundamental choice arises concerning whether to provide live or dry food sources and how frequently to deliver food. Live food, such as *Artemia salina*, Daphnia spp., and chironomid larvae (Chironomidae), offers high nutritional value and can stimulate natural predatory behaviors, thereby providing a more ecologically relevant feeding experience (Chuan et al., 2003; Avramović et al., 2024; Bogut et al., 2007). In contrast, dry foods, including commercial fish pellets, offer standardized nutrition, drastically reduce the chance of food infection and eliminate the need for live food cultures (Žák et al., 2022).

To date, only two dry foods, Otohime C1 (McKay et al., 2022) (Marubeni Nisshin Feed Co., Ltd., Japan) and Biomar INICIO (Žák et al., 2020) (Biomar A/S, Denmark) have been comprehensively tested in *N. furzeri* and reported in the literature. In the present study, we also tested JBL PRONOVO BEL GRANO XS (JBL GmbH & Co. KG, Germany). Although dry food is gradually being adopted by research laboratories, bloodworms remain the predominant food choice for the majority of researchers. This disparity underscores a critical gap: the lack of comparative data on how different dry foods, varying in macronutrient composition, interact with feeding frequency to shape physiological outcomes.

Feeding frequency is another critical aspect of managing the nutritional intake of *N. furzeri.* Infrequent feeding, such as once or twice a day, with gaps of >16 h between the evening and the morning feeding, can promote intermittent fasting, potentially affecting lifespan outcomes (Longo et al., 2021) while excessive feeding can accelerate aging, reduce fertility, or even shorten lifespan, often in sex-specific ways (McKay et al., 2022).

The challenges of manual feeding, including labor intensity, human error, and scalability limitations, have spurred the development of automated systems for laboratory fish husbandry (McKay et al., 2022; Lange et al., 2021; Doyle et al., 2017). However, existing solutions often suffer from high costs, technical complexity, or unreliable precision, hindering their widespread adoption. This study addresses these limitations by introducing a novel, cost-effective, and modular automated feeding system designed specifically for *N. furzeri* aquaria. The system developed in this study eliminates battery dependence, accommodates diverse dry food types, and enables high-frequency feeding (up to 36 times daily), mimicking the continuous food availability observed in natural habitats (Terzibasi Tozzini and Cellerino, 2020; Polačik and Reichard, 2010).

In this study, we introduce a new, practical, cost-effective, and adaptable automatic feeding system designed for standard *Nothobranchius furzeri* aquaria. We hypothesized that diet composition and feeding frequency may influence growth, lifespan, and reproduction in N. furzeri, potentially in a sex-specific manner. Using this platform, we conducted a comprehensive, multi-parametric analysis of how diet composition (high-fat/high-protein vs. low-fat/low-protein dry foods, and live bloodworms) and feeding frequency (automated high-frequency vs. manual twice-daily) influence key physiological parameters including growth, lifespan, and reproductive performance.

Our study contributes significantly to understanding potential diets for this crucial model organism and advances the development of the killifish as a high-throughput model, allowing scalable intervention.

## 2 Materials and methods

### 2.1 Fish husbandry

All animals used in this study represent the short-lived *N. furzeri* strain GRZ (Gonarezhou). Fish were maintained in the killifish facility at the Center for Anatomy & Cell Biology (Medical University of Vienna) under license BMBWF66.009/0130V/3b/2018 (Austrian Federal Ministry of Education, Science and Research) following standardized protocols as previously described (Kabiljo et al., 2019). Aquaria water parameters were maintained at: pH: 7–8; KH: 3–5; and temperature 26 °C–28 °C. Ten percent of the aquarium water in each system was replaced every day. Fish were raised under a 12-h light/dark cycle.

#### 2.1.1 Embryo incubation and hatching

Embryos collected as described in Section 2.6.2 were maintained in Petri dishes in an incubator at 28 °C from the day of collection until day 10, when eye pigmentation first became visible. Embryos were kept in Petri dishes at a maximum density of 70 embryos per dish, in 30 mL of aquarium water. The water was changed daily, and dead embryos were removed during each water change. Daily water changes were performed to maintain water quality and reduce the accumulation of waste, microbial growth, and decomposing material from non-viable embryos.

From day 11, embryos with pigmented eyes were transferred to Petri dishes containing three layers of Whatman paper soaked with 3% humic acid solution (Sigma-Aldrich). Petri dishes were checked and hydrated daily. Embryos were allowed to develop under these conditions in the incubator for 15–25 days, until they reached the golden-eyed stage.

For hatching, golden-eyed embryos were collected individually with forceps and transferred in groups of approximately 50 embryos to the bottom of a 50 mL Falcon tube filled with aquarium water. The water was replaced twice with fresh aquarium water. Finally, the tube was filled with a final volume of 30 mL with aquarium water and incubated at room temperature (RT; 23 °C) without a lid. Hatching occurred within 10–30 min.

#### 2.1.2 Larvae and juvenile fish

Newly hatched larvae were collected and transferred to glass tanks (40 × 20 cm) containing a 2 cm layer of humic acid solution prepared in aquarium water (0.5 g/L; Sigma-Aldrich Cat. No. 53680-10G). Tanks were maintained at 26 °C–28 °C, with a 12-h light/dark cycle and constant aeration. Every day, half of the solution in each hatching tank was removed and replaced with fresh aquarium water without additional humic acid. This gradual replacement progressively diluted the humic acid solution and acclimated larvae to standard aquarium water. During each water change, the bottom of the tanks was cleaned to remove shell fragments and leftover food. Seven days post hatching larvae were moved into the overflow system to 37 × 25 × 16 cm aquaria, with groups of 20–30 larvae per tank. Fish were kept in these conditions for 2 weeks.

#### 2.1.3 Adult fish

At 3 weeks of age, after sex differentiation occurred, male and female fish were separated into different tanks. Male fish were housed individually in 2.8 L tanks, while adult females were kept in groups of 6–8 in 40 × 25 × 25 cm, 25 L group tanks. Males were housed individually to allow individual monitoring of growth and survival, whereas females were group-housed together with one male to allow breeding and egg collection. For female experimental groups, each dietary condition was represented by multiple independent tanks. Specifically, the number of female tanks and individual females per group were as follows: manual bloodworms, 4 tanks/32 females; manual Biomar, 2 tanks/13 females; manual Otohime, 2 tanks/13 females; manual JBL, 2 tanks/9 females; automatic Biomar, 4 tanks/31 females; automatic Otohime, 4 tanks/30 females; and automatic JBL, 3 tanks/22 females. Because females were group-housed for breeding and egg collection, individual females within the same tank may share tank-level environmental and social effects. Therefore, both individual sample size and tank-level biological replication are reported where applicable. At week 4 one male from the single tanks was introduced in each group tank together with females and kept for breeding. As females died over time, they were replaced from other tanks to maintain a density of 6-8 females per group tank.

### 2.2 Automatic feeding system design and installation

The automatic feeding system was designed as a modular, motor-driven bar positioned above a row of standard *N. furzeri* tanks. The system carries removable dry-food tubes aligned with individual tanks, allowing synchronous food delivery during bar rotation. Detailed component specifications, catalog numbers, assembly steps, and 3D-printing information are provided in the Supplementary Technical Protocol and Supplementary Material adaptor. stl.

#### 2.2.1 Mechanical assembly

The main feeding unit consisted of an L-shaped aluminum bar carrying removable food-tube holders positioned according to tank spacing. Each food tube contained dry food and had calibrated openings that allowed gravity-driven food release during rotation. The bar was mounted on the aquarium scaffold using custom frame holders and connected to a 28BYJ-48 stepper motor through 3D-printed adaptors and ball bearings, allowing stable motor-driven rotation above the tanks.

#### 2.2.2 Electrical control

Each stepper motor was connected to a ULN2003 driver board and controlled by an Arduino microcontroller. Multiple motor-driver units could be connected to the same Arduino to allow synchronized rotation of several feeding bars. Because all motors connected to the same Arduino received the same commands, individual motor programming was not used; instead, the amount of food delivered to each tank was adjusted mechanically by modifying the size and/or number of tube openings.

#### 2.2.3 Aquarium lid modification

Tank lids were modified with openings positioned below the food tubes to allow food to enter the corresponding tank during feeder rotation. The openings were covered with a grid to prevent fish escape while allowing complete food deposition into the tank.

### 2.3 Rotation control

Feeder rotation was controlled using Arduino scripts provided in Supplementary Material feeding. ino. The program was designed to perform two types of movements: full rotations, used to dispense food into the tanks, and short alternating partial rotations, used to shake the tubes between feeding events and prevent food aggregation. During each feeding event, the motor performed two full rotations, one clockwise and one anticlockwise.

Feeding frequency was adjusted by modifying the interval between full-rotation feeding events. The shortest interval used in the experiment was 20 min, corresponding to 36 feeding events per day during the 12-h light phase (12 light hours divided by 20-min intervals = 36 feeding events). Lower feeding frequencies, including 12 and 24 feeding events per day, were obtained by increasing the interval between full-rotation events, as summarized in Supplementary Table S3. Between feeding events, partial shaking rotations were performed every 5 min.

Each Arduino was connected to a digital timer synchronized with the room 12-h light/12-h dark photoperiod. The timer switched on the Arduino, and therefore the feeding program, during the light phase and off during the dark phase. This ensured that food was delivered only during daytime and prevented nocturnal food delivery. Detailed Arduino settings, timing parameters, and code information are provided in the Supplementary Technical Protocol and Supplementary Material feeding. ino.

### 2.4 Fish feeding and feeding programs

During the first week post hatching, all fish were fed *ad libitum* with *Artemia salina* (Sanders) twice per day. Starting on day 7, fish were divided into seven different feeding groups. Three groups were automatically fed with Biomar INICIO Plus, Otohime C1, and (JBL) dry foods, respectively. Automatic feeding was performed only during the light phase and varied according to fish age and housing condition, with feeding frequencies ranging from 12 to 36 feedings per day. The complete feeding regimen, including age phases, feeding frequency, rotations per feeding event, tube openings, and the amount of food delivered per fish per day, is summarized in Supplementary Table S3. Food tubes were filled with 10 g of dry food and refilled weekly every Monday to restore the amount to 10 g. No moisture accumulation, mold growth, or food spoilage was observed in the tubes during the experimental period. The programmed 5-min shaking intervals effectively prevented food aggregation. The remaining four groups were fed manually with Biomar, Otohime, JBL, or chironomid larvae (bloodworms). Manual feeding was performed twice daily, around 10:00 and 15:00. For dry-food groups, each manual feeding provided half of the total daily food amount delivered to the corresponding automatically fed group, so that the total daily dry-food amount was matched between manual and automatic feeding conditions. Bloodworms were provided at a fixed standard amount exceeding the maximum amount that an individual fish or group of fish could consume within 10 min, plus an additional 2–5 bloodworms per feeding.

### 2.5 Food delivery tests

Food delivery precision was quantified using a reduced feeder setup coupled to an Arduino-compatible load-cell scale. Three dry foods, Biomar, Otohime, and JBL, were tested using three tube-opening diameters: 1.5 mm, 2.0 mm, and 2.5 mm. For each food/opening combination, three independent tubes were used as technical replicates, resulting in 27 total tests (3 foods x 3 openings x 3 tubes replicates).

Before each test, tubes were filled with exactly 10 g of dry food and positioned in the feeder. During each feeding event, the motor performed two full rotations, one clockwise and one anticlockwise, allowing food to be released onto the scale. After each feeding event, the scale signal was recorded and later converted to grams. Calibration was performed by measuring the total amount of food dispensed at the end of each test with an independent scale and using this value to convert the recorded digital scale signal into grams.

For each food/opening combination, food delivery was calculated from 240 feeding events, corresponding to 480 individual food drops. The average amount of food delivered per feeding event was calculated and used to compare delivery across food types and tube-opening sizes. Delivery consistency over time was assessed by comparing repeated sets of feeding events within each test. Detailed information on the testing frame, wiring, software settings, serial monitor acquisition, calibration procedure, and product sources is provided in the Supplementary Technical Protocol.

### 2.6 Data collection of physiological parameters

#### 2.6.1 Fish growth

All fish in each group were measured at 4-week intervals, from week 6 to week 26. For weight assessment, fish were captured with a net, gently blotted dry with paper towels while in the net, and then placed in a water-filled container on a scale. Length was determined by photographing each fish alongside a reference ruler in the same container. Images were calibrated using the ruler as a scale reference, and fish length was measured using Fiji ImageJ. Because growth was monitored repeatedly over time in the same experimental groups, longitudinal growth curves were used to visualize changes in body weight and length across age. Statistical comparisons reported for growth endpoints were performed at defined time points rather than by treating all repeated measurements across time as independent observations. Effect estimates reported in Supplementary Table S1 were calculated at 18 weeks of age, a time point selected because fish were adult, growth differences were established, and sufficient measurements were still available across groups before later survival-related reductions in sample size.

#### 2.6.2 Breeding and embryos collection

Females in group tanks were bred once per week, every week, from the sixth week of life to week 20. To initiate the breeding event, a filter tip box (10 × 8 × 6 cm) half filled with sand (ø <0.2 mm) was placed on the bottom of the tanks. The sand boxes were introduced in the morning after the manual feeding and removed in the afternoon just prior to the second manual feeding, providing a breeding window of almost 5 h. Embryos were collected by removing the boxes from the tank and pouring the sand through a strainer (1 mm grid width). Embryos retained in the strainer were transferred to a petri dish filled with aquarium water. Embryos from female tanks belonging to the same diet and feeding-regimen group were collected together during each weekly breeding session. Thus, embryos were pooled by treatment group at the time of collection, and the identity of the individual source tank was not retained. Consequently, reproductive outcomes represent treatment-level pooled values for each diet and feeding-regimen combination over time. Because tank-level replication was not preserved for these reproductive measurements, these data were interpreted descriptively as treatment-level reproductive profiles rather than as tank-level inferential comparisons.

#### 2.6.3 Embryos counting and survival

After collection, non-viable and malformed embryos were counted and discarded. Fertilized, morphologically normal embryos were enumerated and transferred to petri dishes containing 35 mL of reverse osmosis (RO) water supplemented with 1% methylene blue (Sigma-Aldrich, CAS: 122965-43-9), with a maximum density of 50 embryos per dish. Dishes were incubated at 28 °C in a ventilated incubator. Embryos were monitored daily, and the solution was replaced once daily with fresh RO water +1% methylene blue. Non-viable embryos were counted and removed during each replacement. Between days 9 and 15, when most embryos reached the black-eyed or diapause II stage, the count was recorded as the surviving embryo fraction.

#### 2.6.4 Fish lifespan monitoring

Fish survival was monitored daily during morning inspections. Deceased fish were removed from the tanks and recorded on the day of death. Survival time was calculated as the age of each fish at death, expressed in days post-hatching and then converted to weeks for graphical representation. Daily mortality records were subsequently entered into Excel at 2-week intervals for data organization and visualization. Lifespan was assessed for the same fish used in growth and egg production measurements, excluding breeding males housed in female group tanks.

### 2.7 Statistical analysis

Statistical analyses were performed using GraphPad Prism. Pairwise comparisons were performed using t-tests, while multiple comparisons were analyzed by ANOVA. The specific type of ANOVA (one-way or two-way) and the *post hoc* tests used are indicated in the corresponding figure legends. Survival curves were generated using the Kaplan–Meier method and compared between groups using the log-rank (Mantel–Cox) test. Survival analyses were performed using the daily mortality records collected for each individual fish. Exact p-values for growth and survival analyses are provided in Supplementary Tables S1, S2, respectively, while median survival values are summarized in the physiological overview table. For growth analyses, body weight and length were measured repeatedly over time and are presented as longitudinal growth curves. To avoid treating repeated time points from the same experimental groups as independent observations, statistical comparisons reported in Supplementary Table S1 were performed at defined age points, and effect estimates were calculated using raw measurements at 18 weeks of age. Reproductive outcomes were analyzed descriptively because embryos from tanks receiving the same diet and feeding regimen were pooled at collection, and tank-level identity was not retained. Therefore, these data represent treatment-level reproductive profiles over time, and no tank-level inferential statistics were performed for egg production, embryo survival, or reproductive score.

### 2.8 Graphs, videos, and images

Graphics and drawings were created using GIMP and PowerPoint. Graphs were generated using Excel, and videos were edited using Videopad.

## 3 Results

### 3.1 Design, implementation and validation of a high-precision automated feeding system

A precise and reliable feeding method is essential for maintaining consistent conditions among specimens and ensuring reproducible experimental results across animal groups. Automated feeding systems, especially robotic designs, offer significant advantages over manual methods by improving precision and minimizing human-introduced biases (e.g., variability in users, food distribution, or feeding schedules). Furthermore, automated systems are highly scalable, capable of simultaneously feeding numerous fish, a critical feature when time intervals between feeding events become a limiting factor. To date, various automatic feeding systems have been developed, each with its own set of advantages and limitations, including high costs, complex infrastructure, unreliable precision, intricate design, and frequent maintenance requirements (McKay et al., 2022; Lange et al., 2021; Doyle et al., 2017). To address these limitations, we developed a cost-effective, battery-free, modular, and easily refillable automatic food dispenser for standard N. furzeri aquaria. The system was designed to provide synchronized feeding across multiple tanks, flexible adherence to custom feeding schedules, reliable delivery of small food quantities, low construction cost (ca. 500 euros for 100 tanks), and compatibility with powdered, granular, or pelleted dry fish food.

The automated feeder consists of a motor-driven bar carrying removable dry-food tubes with variable-size openings, positioned above a row of tanks and aligned with individual aquaria (Figures 1A,B). During each feeding event, the bar rotates and dispenses food synchronously into the tanks below (Figure 1C; Supplementary Video S2). The system can be installed above standard individual tank rows by attaching the feeder frame to the aquarium scaffold (Figure 1D; Supplementary Video S1). Detailed assembly instructions and component specifications are provided in the Materials and Methods, in the Supplementary Technical Protocol, and in the step-by-step visual assembly guide shown in Supplementary Figure S1.

**FIGURE 1:**
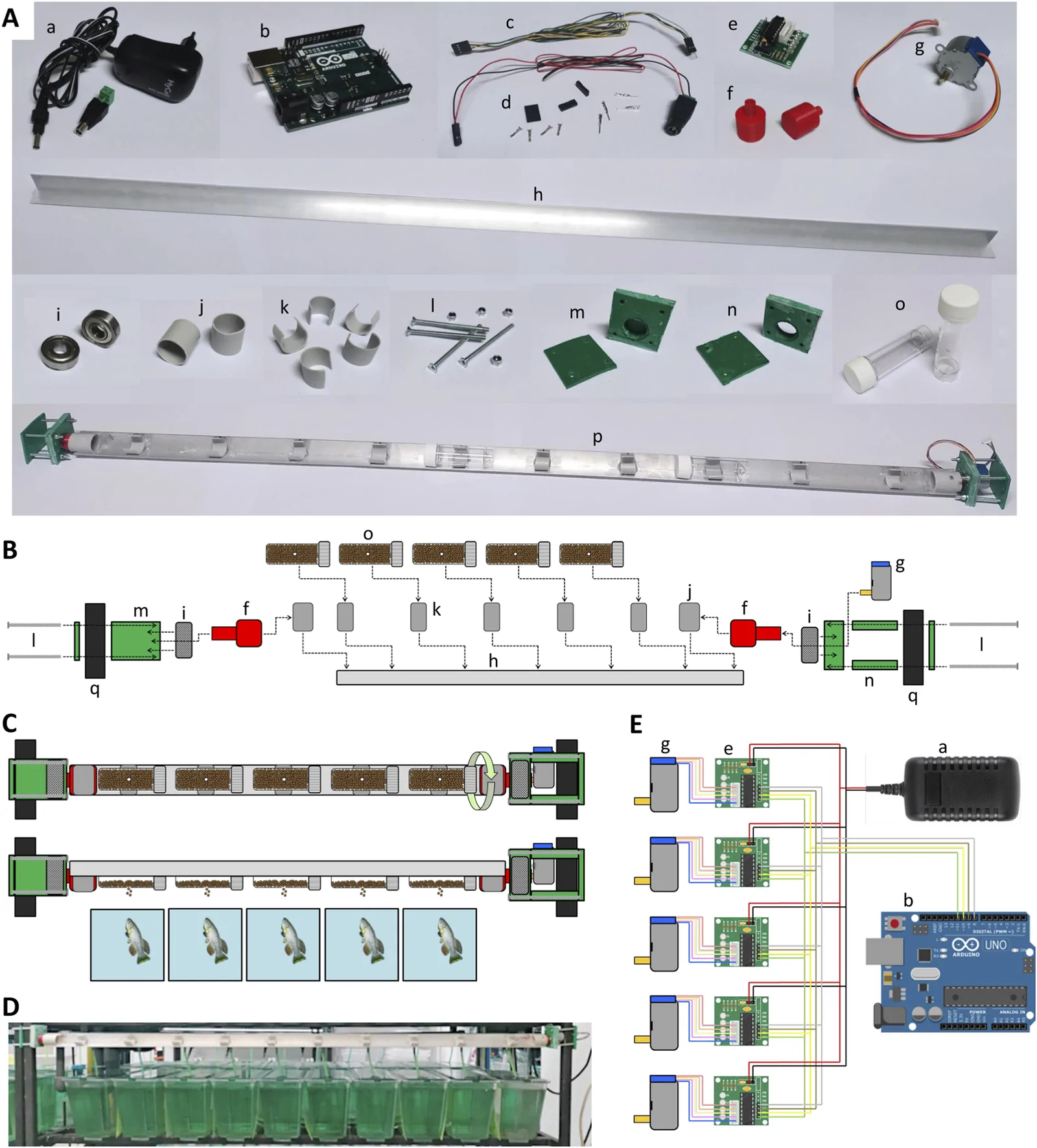
*Nothobranchius furzeri* Automatic Feeding System. **(A)** Components for feeder assembly. **(B)** Automatic feeder assembly schematic. Component reference letters are consistent with the previous panel. **(C)** Feeding-event schematic illustrating synchronous food delivery into the tanks below during motor-driven bar rotation. **(D)** Front view of the automatic feeder installed above a row of individual 2.8 L tanks. **(E)** Circuit diagram of the automatic feeder. Detailed component specifications, catalog numbers, assembly steps, and 3D-printing information are provided in the Supplementary Technical Protocol, Supplementary Material adaptor. stl, and the step-by-step visual assembly guide shown in Supplementary Figure S1.

Bar rotation is controlled by an Arduino microcontroller (Figure 1E; Supplementary Video S3), and multiple units can be connected in parallel to allow synchronized feeding across tank rows. Because all motors connected to the same Arduino execute the same command, food delivery to individual tanks is adjusted mechanically by modifying the number and/or size of tube openings rather than by programming motors independently.

In contrast to manual feeding, which is usually performed twice per day, the automated feeder was programmed to deliver food repeatedly throughout the light phase, up to 36 times per day, corresponding to one feeding event every 20 min (Figures 2A,B).

**FIGURE 2:**
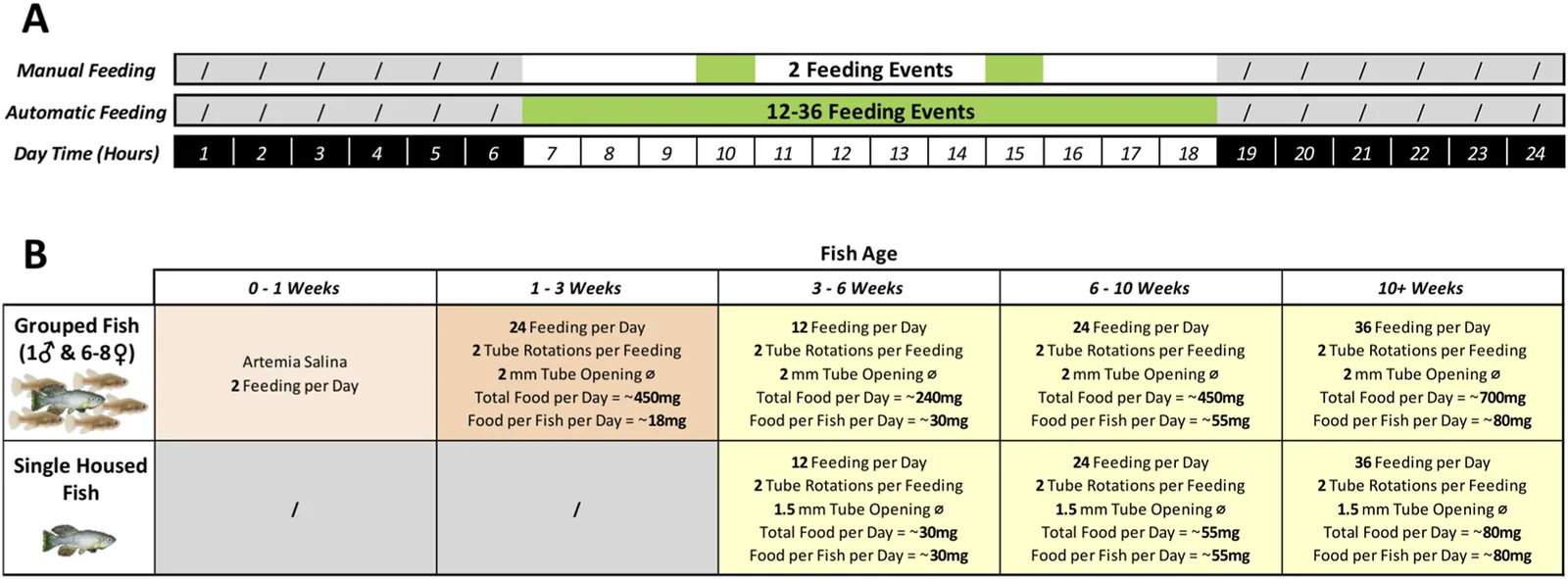
Fish Feeding Schedule. **(A)** Schematic representation of automatic (12–36 feedings per day) and manual (twice per day) feeding schedules. Black and white boxes represent daytime, while green highlights indicate feeding windows. Both manual and automatic feeding deliver the same total amount of food (in grams) throughout the day, with the distribution varying based on the number of feedings. **(B)** Dry Food Automatic Distribution by Fish Life Stage. Illustration of the increasing amount of food delivered to fish as they grow, resulting in a greater number of feedings per day. Single-housed and group-housed fish receive an equal amount of food per fish per feeding event. Grouped fish are housed in groups consisting of 6–8 females and 1 male. Detailed feeding parameters are reported in Supplementary Table S3.

#### 3.1.1 Precision and reliability of gravity-fed food dispensation in automated feeders

Food dispensation depends on gravity-driven release of granules or pellets through tube openings during rotation. Because delivery may be influenced by food shape, granule size, food density, and opening diameter, we tested the precision and reliability of food delivery using a reduced feeder setup coupled to an Arduino-compatible load-cell scale with a precision of 0.001 g (Figures 3A,B). Three dry food types and three tube-opening diameters were tested: Biomar, Otohime, and JBL with 1.5 mm, 2.0 mm, and 2.5 mm openings.

**FIGURE 3:**
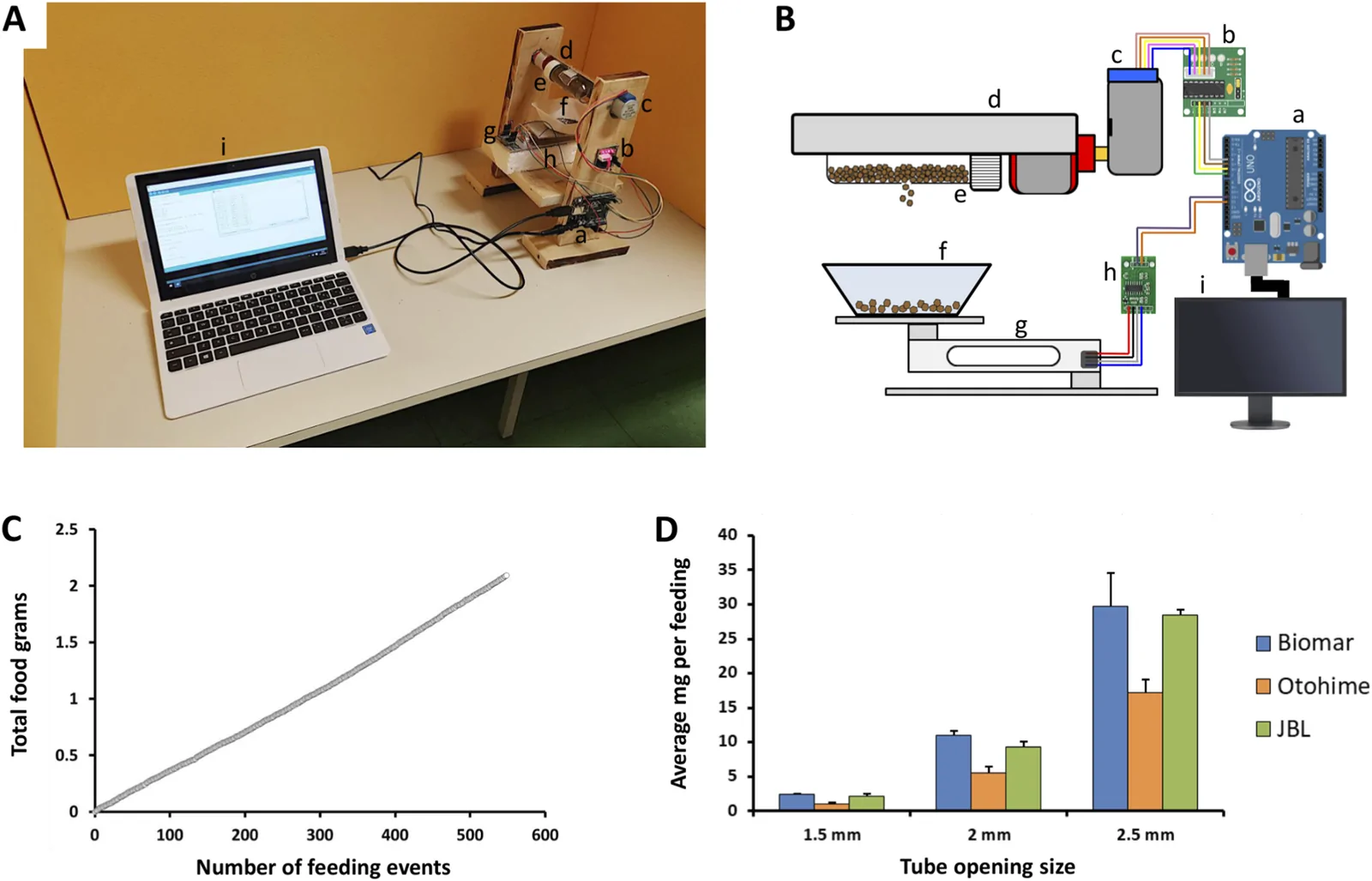
Automatic Feeder Food Delivery Tests. **(A)** Setup used to assess food delivery kinetics and accuracy. **(B)** Schematic representation of the testing setup, in which a reduced automated feeder releases food onto a high-precision load-cell scale connected to an Arduino-compatible acquisition system. **(C)** Representative cumulative food-delivery trace over repeated feeding events, showing consistent food release over time. **(D)** Average food delivered per feeding event for three dry foods, Biomar, Otohime, and JBL, using three tube-opening diameters: 1.5 mm, 2.0 mm, and 2.5 mm. Food delivery increased with opening diameter, while food-specific differences likely reflected granule size, shape, and density. Detailed testing setup, calibration, and data-acquisition procedures are provided in the Supplementary Technical Protocol.

The amount of food dispensed remained consistent over time, and no system failures, opening occlusions, or abnormal food delivery events were observed during the tests (Figure 3C). Food delivery increased with larger tube openings, and each food type showed a distinct delivery profile, likely reflecting differences in granule size, shape, and density (Figure 3D). Overall, the feeder achieved reproducible and tunable food delivery, particularly with smaller opening sizes. The system could deliver as little as approximately 1 mg of food per feeding event, demonstrating sufficient resolution for high-frequency feeding experiments in *N. furzeri*.

Beyond technical validation, the automated feeder was implemented as a biological husbandry platform to test feeding frequency as an experimental variable under controlled dry-food delivery. By distributing the delivered daily dry-food amount across repeated feeding events during the light phase, the system allowed us to compare high-frequency feeding with standard twice-daily feeding and to assess downstream effects on growth, survival, and reproduction.

#### 3.1.2 Physiological responses of fish to automated and manual feeding systems with diverse nutritional profiles

The automated feeders were used to assess the impact of varying diets and feeding regimens on key physiological parameters in fish. A total of four different foods with varying nutritional properties were tested (Table 1A):

**TABLE 1:** (A) Nutritional Composition of Tested Foods. Macronutrient composition of the selected dietary options provided to the fish. Nutritional values for dry foods are sourced from the manufacturer’s online data sheet when available (Biomar Inicio Plus, Otohime C1) or from comparable product data where direct labels were not available (JBL), while the values for bloodworms are estimated based on the publication by Zak et al., 2020. (B) Fish feeding experimental plan. Fish were divided into groups and subgroups according to sex, housing condition, feeding regimen, and diet. Males were housed individually, whereas females were accommodated in groups of 6–8 per tank together with one male. Data were collected for each individual dietary group and, where indicated, also analyzed as aggregated feeding-regimen groups. Fish and tank numbers for each group and diet are reported in the corresponding cells. For individually housed males, the number of fish corresponds to the number of independent tanks. For group-housed females, both the number of individual fish and the number of independent tanks are reported.

	Crude protein	Crude lipid	Ash	Estimated calories pro 100g
(A)				
**Biomar INICIO Plus 0.8**	*56.0%*	*18.0%*	*11.3%*	*521*
**Otohime C1**	*50.0%*	*10.0%*	*17.0%*	*450-500*
**JBL PRONOVO BEL GRANO XS**	*38.0%*	*6.0%*	*7.0%*	*380-430*
**Bloodworms**	*53.8%*	*5.2%*	*12.3%*	*100-130 (wet) 450-550 (dry)*

	Single housed male		Group housed female	
(B)				
**Food Type**	- Group 1 - Automatic Feeding (12-36 Times per Day)	- Group 2- Manual Feeding (Twice per Day)	- Group 3 - Automatic Feeding (12-36 Times per Day)	- Group 4 - Manual Feeding (Twice per Day)
**Biomar**	*(n=15)*	*(n=15)*	*(n=31, tanks=4)*	*(n=13, tanks=2)*
**Otohime**	*(n=14)*	*(n=13)*	*(n=30, tanks=4)*	*(n=13, tanks=2)*
**JBL**	*(n=11)*	*(n=18)*	*(n=22, tanks=3)*	*(n=9, tanks=2)*
**Bloodworms**	*/*	*(n=38)*	*/*	*(n=32, tanks=4)*

Color coding is used only to improve readability. Food names are color-coded consistently across the manuscript figures and tables: Biomar, blue; Otohime, orange; JBL, green; bloodworms, red. Shaded headers indicate experimental housing/feeding categories and do not represent statistical significance.

Biomar INICIO Plus: a high-fat and high-calorie pelleted food.

Otohime C1: a granular food with medium-high protein, fat, and calorie content.

JBL: a low-protein, low-fat, and low-calorie granular food.

Bloodworms: live and frozen food with a fat content similar to JBL PRONOVO and a protein content similar to Biomar and Otohime food (Žák et al., 2020).

Biomar, Otohime, and JBL foods were tested both with automatic feeders and with manual feeding, whereas bloodworms were exclusively tested manually, as automatic feeders cannot dispense non-granular feeds.

The study encompassed four major groups of fish:

Group 1: Single-housed male fish receiving dry food via automated delivery 18 to 36 times per day, totaling 30–80 mg per fish per day.
Group 2: Single-housed male fish receiving either dry or live bloodworm food via manual delivery twice daily, totaling 30–80 mg per fish per day.
Group 3: Group-housed female fish receiving dry food via automated delivery 18 to 36 times per day, totaling 30–80 mg per fish per day.
Group 4: Group-housed female fish receiving either dry or live bloodworm food via manual delivery twice daily, totaling 30–80 mg per fish per day.

Fish fed dry food were further divided into subgroups based on the three different dry food types tested (Biomar, Otohime, JBL) (Table 1B). Fish were grown and maintained under these feeding conditions from their second week of life. Various parameters, such as lifespan, fish growth, fish size, egg production, and egg survival, were assessed and compared across the different groups. Egg production and survival were evaluated weekly starting from the sixth week of age, while fish size and growth rate were assessed every 4 weeks, also commencing from the sixth week.

### 3.2 Automated high-frequency feeding promotes accelerated fish growth independent of diet type or sex

Fish fed automatically at high frequency (up to 36 times per day) exhibited significantly faster and greater growth compared to those fed manually twice daily, regardless of sex (Figure 4A). In the group receiving dry food automatically up to 36 times daily, both body weight and length increased more rapidly over time compared to the group fed dry food only twice daily. Additionally, maximum body weight and length at 26 weeks of age were significantly greater in the high-frequency feeding group. Conversely, fish fed bloodworms showed similar length growth to those fed dry food automatically, for both sexes, but maintained significantly lower body weight compared to the automatically fed group. Moreover, the type of dry food used generally did not affect growth, as no statistically significant differences were observed among the three dry food groups when fish received the same number of daily feedings. The only exception was the group fed JBL twice daily, which exhibited significantly slower growth and smaller final size compared to the other groups (Figure 4B). Thus, growth rates were primarily influenced by feeding frequency, rather than the specific diet type or total food quantity. These results suggest that increased feeding frequency may increase effective food intake and/or nutrient uptake, thereby promoting accelerated growth across the fish’s lifespan.

**FIGURE 4:**
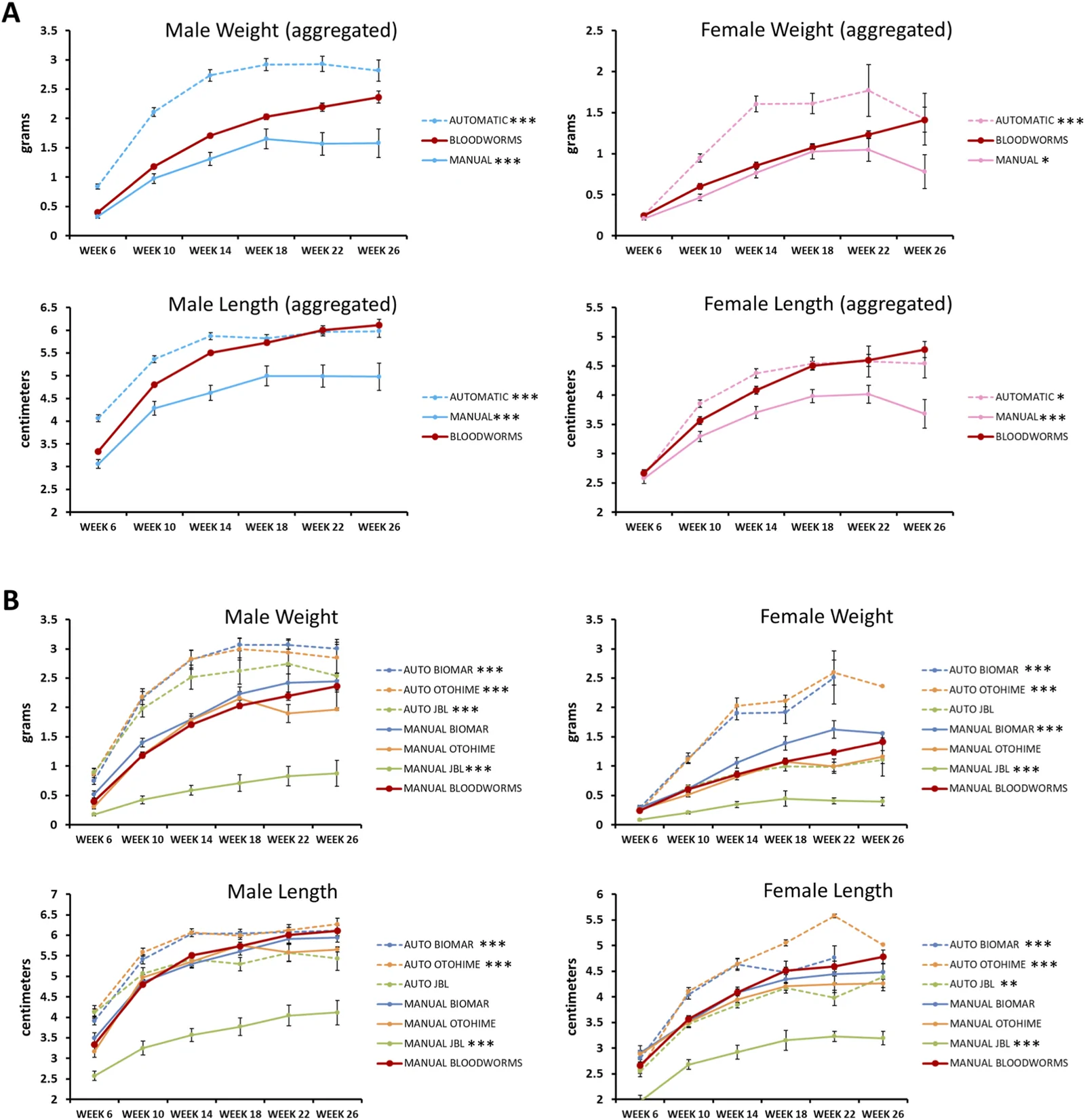
Growth Rate in Different Dietary Groups. **(A)** Aggregated data showing the increase in weight and length over time for male fish (blue lines) and female fish (pink lines) fed automatically up to 36 times per day (dashed lines) or manually twice per day (solid lines). Dark red lines represent bloodworms, serving as a reference. **(B)** Individual Fish-Group Data. Curves representing the growth of specific fish groups, fed with particular food types (legend at the bottom), either manually (solid lines) or automatically (dashed lines). Male fish are shown on the left and female fish on the right. Statistical differences in body weight and length are indicated as ns, not significant; *, p < 0.05; **, p < 0.01; ***, p < 0.001. Detailed statistical results are reported in Supplementary Table S1. Group sizes and tank numbers are reported in Table 2B. Detailed statistical results are provided in Supplementary Table S1.

### 3.3 Feeding regimen and diet composition are associated with sex-specific survival patterns in individually housed males and group-housed females

Lifespan analysis revealed distinct survival trends between individually housed males and group-housed females, particularly in the interaction between feeding regimen and survival. Because males and females were maintained under different housing conditions, these survival patterns should be interpreted as differences observed between individually housed males and group-housed females, rather than as effects attributable exclusively to biological sex. Male survival did not differ statistically across feeding groups, regardless of whether fish were fed automatically or manually, or received dry food or bloodworms (Figure 5A). To identify overarching patterns, we pooled data, comparing all automatically fed males with manually fed males and the bloodworm-fed group. Unexpectedly, males fed dry food twice daily exhibited a median lifespan statistically indistinguishable from those receiving more frequent feedings (Figure 5B). This result contradicts the established paradigm that higher caloric intake reduces lifespan, whereas calorie restriction promotes longevity. This discrepancy is further highlighted by the observation that automatically fed males exhibited significantly larger body sizes (Figure 4A). In contrast, female lifespan varied markedly across feeding groups. Females fed manually showed no significant differences in lifespan among dry food groups and the bloodworm group, suggesting that food type did not influence survival at the tested dosages. Strikingly, females subjected to automatic feeding exhibited significantly shorter lifespans than those fed bloodworms (Figure 5C). Notably, high-frequency feeding revealed a strong correlation between food caloric content and female lifespan, at least until the median survival point. Fish receiving higher-calorie diets exhibited shorter lifespans, particularly in the Biomar and Otohime groups, which had the shortest survival. The JBL group, however, showed lifespans more comparable to bloodworm-fed fish.

**FIGURE 5:**
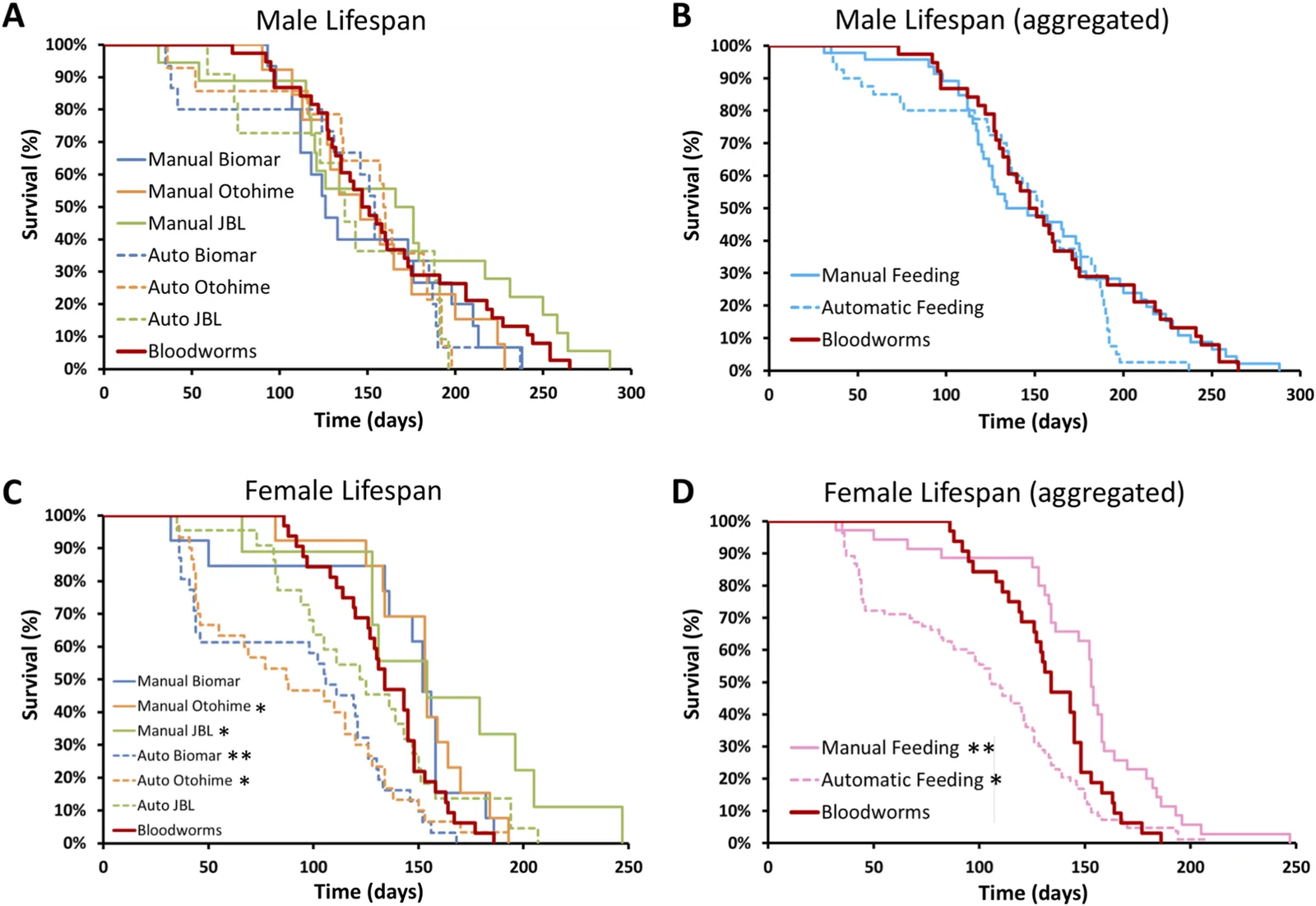
Survival Curves of Fish Fed with Different Dietary Foods and Regimens. **(A)** Lifespan curves for each specific male fish group fed with a particular food type, either manually (solid lines) or automatically (dashed lines). Group sizes: Bloodworms (n = 38), Manual Biomar (n = 15), Manual Otohime (n = 13), Manual JBL (n = 18), Automatic Biomar (n = 15), Automatic Otohime (n = 14), and Automatic JBL (n = 11). **(B)** Aggregated male lifespan data for fish fed manually twice per day (solid lines, n = 46) or automatically up to 36 times per day (dashed lines, n = 40). The dark red line represents bloodworms and serves as a reference. **(C)** Lifespan curves for female fish fed with each specific diet, either manually (solid lines) or automatically (dashed lines). Group sizes and, where applicable, independent tank numbers are reported in Table 2B. **(D)** Aggregated female lifespan data for fish fed manually twice per day, n = 35 females from 6 tanks, or automatically up to 36 times per day, n = 83 females from 11 tanks. Bloodworms, n = 32 females from 4 tanks, are shown as a reference. Statistical differences between survival curves were assessed using the log-rank (Mantel–Cox) test. Significance is indicated as ns, not significant; *, p < 0.05; **, p < 0.01; ***, p < 0.001. Detailed statistics and median survival values are reported in Supplementary Table S2.

Critically, aggregated data revealed that females fed up to 36 times daily had significantly shorter lifespans than those fed bloodworms, while those receiving dry food twice daily lived significantly longer (Figure 5D). While this aligns with established literature on calorie restriction and lifespan (Hwangbo et al., 2020; Dorling et al., 2020; Kane et al., 2018; Mitchell et al., 2016), it contrasts with previous Nothobranchius studies, where dietary regimens did not significantly affect female survival (McKay et al., 2022).

Finally, it is important to note that survival differences were most pronounced up to the median lifespan; beyond this point, lifespans converged toward a similar maximum age across all groups.

### 3.4 Pooled reproductive profiles suggest associations between diet, feeding frequency, and reproductive timing

Egg production and embryo survivability presented a complex interplay, with pooled reproductive profiles suggesting associations between feeding frequency, diet type, fecundity, and fertility (Figure 6).

**FIGURE 6:**
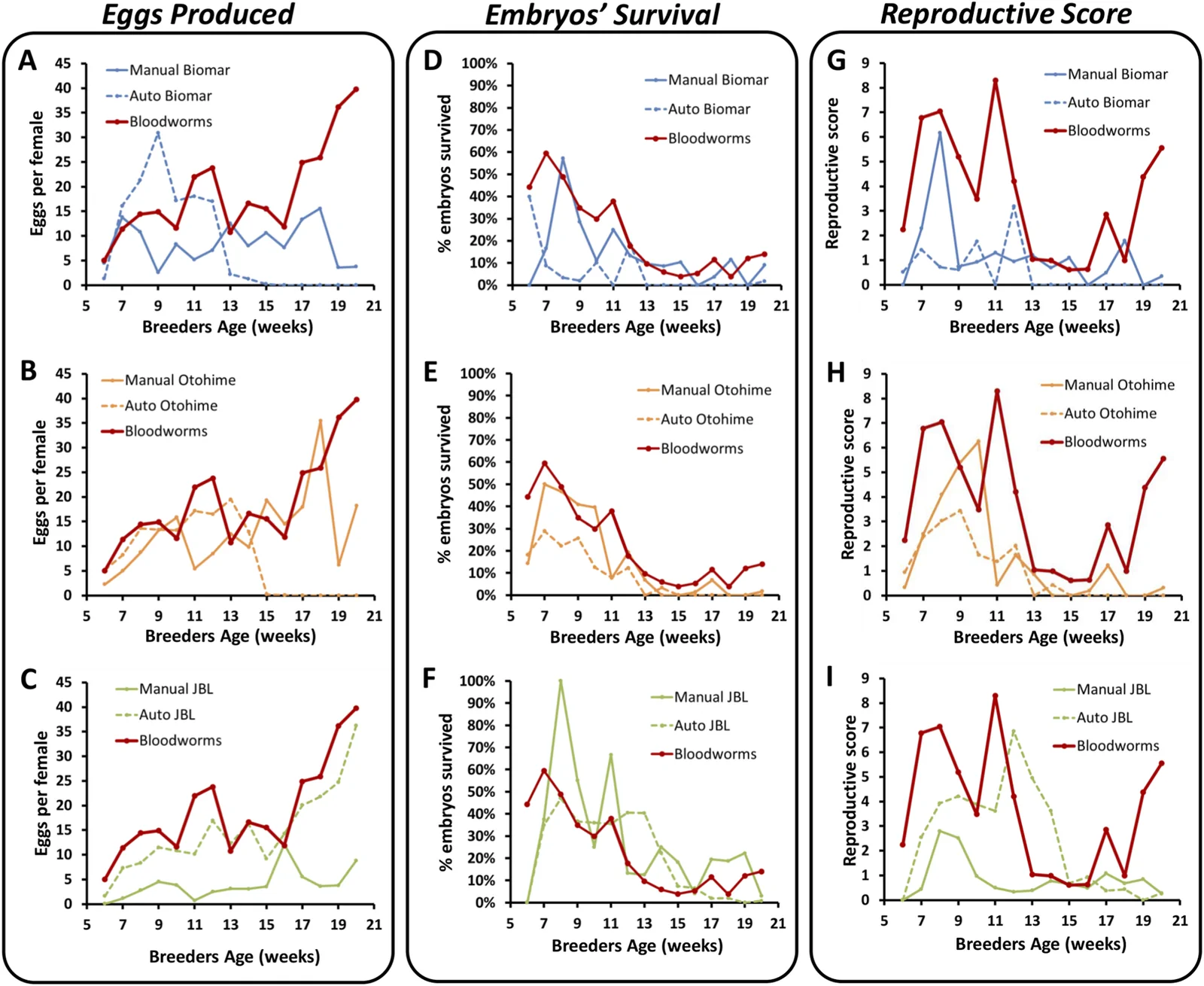
**(A–C)** Egg production per female over time for Biomar, Otohime, and JBL diets under automatic (dashed lines) and manual (solid lines) feeding. Bloodworms (red) are shown as a reference across all panels. Automatic feeding was associated with earlier and sharper fecundity peaks in all dry diets, whereas manual feeding produced lower but more sustained egg output. Bloodworms showed the highest pooled egg production and the longest reproductive window. **(D–F)** Embryo survival, expressed as the percentage of embryos reaching pigmentation or diapause II. All dry diets showed rapid declines in fertility, particularly under automatic feeding. Biomar produced the lowest embryo survival, JBL the highest, and Otohime intermediate. Bloodworm-fed fish showed high pooled embryo survival for most of the reproductive period. **(G–I)** Reproductive score, calculated as egg number × embryo survival, integrating fecundity and fertility into a single metric. Bloodworms showed the highest pooled and most sustained reproductive performance across nearly all ages. Dry diets displayed pronounced trade-offs: Biomar exhibited high fecundity but low survival, JBL showed the reverse trend, and Otohime presented intermediate profiles. Automatic feeding was associated with a more compressed reproductive profile, whereas manual feeding was associated with a more prolonged reproductive profile. *Data represent pooled treatment-level reproductive profiles because embryos were pooled by diet and feeding-regimen group at collection; therefore, no tank-level inferential statistics were performed *.

Fecundity, defined as the total number of eggs produced per fish group, was quantified by weekly egg counts within a defined timeframe. Fertility, reflecting the actual reproductive success of a fish group, was assessed by counting fertilized embryos that reached critical survival stages (e.g., pigmentation or diapause 2).

For fish fed automatically, fecundity peaked at 9, 13, and 20 weeks, while fertility (embryo survival) reached its maximum at 6, 7, and 8 weeks for Biomar, Otohime, and JBL diets, respectively. In contrast, manually fed fish exhibited fecundity peaks at 18, 18, 16, and 20 weeks and fertility peaks at 8, 7, 8, and 7 weeks for Biomar, Otohime, JBL, and bloodworms, respectively (Figure 6).

Among the pooled reproductive profiles, bloodworm-fed fish showed the highest values for fecundity, embryo survival, and reproductive score (defined as the product of egg production and survival) across nearly all breeder ages (Figure 6, red lines). Fish fed bloodworms sustained egg production over an extended reproductive window and maintained high embryo survival throughout the experiment, suggesting that this diet supported the most favorable pooled reproductive profile.

In all dry-food groups (Biomar, Otohime, JBL), automatic feeding was associated with early, pronounced peaks in egg production, which declined rapidly over time (Figures 6A–C). These peaks were consistently followed by sharp declines in embryo survival (Figures 6D–F) and reproductive score (Figures 6G–I), suggesting that high-frequency feeding may be associated with an earlier and more compressed reproductive profile. Conversely, manually fed fish exhibited more moderate fecundity but sustained reproductive output over a longer period, indicating that lower feeding frequency was associated with a more prolonged reproductive profile.

The pooled dry-food profiles suggested a trade-off between egg quantity and embryo survival that varied with nutrient composition. Biomar (highest fat and protein content) yielded the highest egg production but the lowest embryo survival, particularly under automatic feeding. JBL (lowest protein/fat content) produced fewer eggs but achieved the highest embryo survival, often matching or surpassing bloodworms during early reproductive weeks. Otohime exhibited intermediate performance, with moderate egg production and embryo survival. This trade-off was directly reflected in the reproductive score: diets optimizing egg quantity compromised embryo survival, while lower-yield diets maintained higher egg quality.

Feeding regimens altered reproductive windows, underscoring the influence of feeding frequency on reproductive timing. Automatic feeding condensed reproduction into a brief, intense early phase, particularly for Biomar and Otohime, which transitioned rapidly from peak egg production to near-infertility. In contrast, manual feeding prolonged the reproductive window by several weeks, producing more stable trends in egg production, embryo survival, and reproductive score. While automatic feeding amplified all observed trends (e.g., higher fecundity peaks, faster fertility declines), the relative ranking of diets remained consistent: bloodworms > Otohime/JBL (metric-dependent) > Biomar. Nutrient composition, rather than feeding frequency alone, appeared to be an important factor associated with reproductive outcomes.

Collectively, each diet and feeding regimen produced a distinct profile of growth, lifespan, and reproduction. No single diet or feeding method universally showed the most favorable profile across all endpoints; rather, each combination was associated with different physiological patterns (Table 2).

**TABLE 2:** (A) Diet Effects on Fish Physiological Parameters Overview. A comprehensive summary of each diet effect on fish lifespan and growth. The table displays the values recorded for each individual food supplied, both manually and automatically, as well as the aggregated values from all automatically fed fish versus all manually fed fish. (B) Diet Effects on Fish Reproductive Health Overview. A comprehensive summary of the effects of each diet on fish fertility and fecundity. The table presents the values recorded for each individual food supplied, both manually and automatically, as well as the aggregated values from all the automatically fed fish versus all the manually fed fish.

			*Lifespan*		*Growth*			
			Median Survival	Max Survival	Body weight gain per week (weeks 6–18)	Maximum body weight gain	Body length gain per week (weeks 6–18)	Maximum body length gain
	(A)							
Male Fish	Automatic Feeding	*Biomar*	22 weeks	33.9 weeks	0.176 g/week	3.072 g	0.296 cm/week	6.114 cm
		*Otohime*	22.8 weeks	28.3 weeks	0.169 g/week	2.995 g	0.288 cm/week	6.27 cm
		*JBL*	19.6 weeks	28 weeks	0.145 g/week	2.741 g	0.245 cm/week	5.572 cm
	Manual Feeding	*Biomar*	18 weeks	34 weeks	0.129 g/week	2.443 g	0.278 cm/week	5.939 cm
		*Otohime*	20.9 weeks	32.6 weeks	0.128 g/week	2.155 g	0.294 cm/week	5.757 cm
		*JBL*	24.4 weeks	41.1 weeks	0.041 g/week	0.876 g	0.182 cm/week	4.115 cm
		*Bloodworms*	21.3 weeks	37.9 weeks	0.118 g/week	2.366 g	0.289 cm/week	6.109 cm
Female Fish	Automatic Feeding	*Biomar*	15.1 weeks	24 weeks	0.114 g/week	2.511 g	0.222 cm/week	4.761 cm
		*Otohime*	12.5 weeks	27.6 weeks	0.127 g/week	2.597 g	0.262 cm/week	5.58 cm
		*JBL*	17.6 weeks	29.6 weeks	0.057 g/week	1.108 g	0.208 cm/week	4.384 cm
	Manual Feeding	*Biomar*	21.7 weeks	26.6 weeks	0.081 g/week	1.628 g	0.211 cm/week	4.482 cm
		*Otohime*	21.9 weeks	27.6 weeks	0.062 g/week	1.157 g	0.203 cm/week	4.262 cm
		*JBL*	22 weeks	35.3 weeks	0.026 g/week	0.446 g	0.156 cm/week	3.228 cm
		*Bloodworms*	19.1 weeks	26.6 weeks	0.062 g/week	1.413 g	0.226 cm/week	4.777 cm

Automatically Fed Male Aggregated average			22 weeks	33.9 weeks	0.165 g/week	2.928 g	0.279 cm/week	5.976 cm
Manually Fed Male Aggregated average			20 weeks	41.1 weeks	0.096 g/week	1.65 g	0.248 cm/week	4.995 cm
Automatically Fed Female Aggregated average			15.1 weeks	29.6 weeks	0.095 g/week	1.77 g	0.229 cm/week	4.577 cm
Manually Fed Female Aggregated average			21.9 weeks	35.3 weeks	0.06 g/week	1.047 g	0.195 cm/week	4.017 cm

		*Fertility and Fecundity*					
		Max Egg per Female produced (in 1 week)	Fish Age at Max Egg production	Max Embryos' Survival (From 1 collection)	Fish Age at Max Embryo Survival	Max Reproductive Score (Egg number*Survival)	Fish Age at Max Fertility / Fecundity Ratio
(B)							
Automatic Feeding	*Biomar*	30.9 eggs	9 weeks	40 %	6 weeks	3.19	12 weeks
	*Otohime*	19.5 eggs	13 weeks	28.9 %	7 weeks	3.44	9 weeks
	*JBL*	36.3 eggs	20 weeks	47.1 %	8 weeks	6.86	12 weeks
Manual Feeding	*Biomar*	15.5 eggs	18 weeks	57.1 %	8 weeks	6.17	8 weeks
	*Otohime*	35.5 eggs	18 weeks	50 %	7 weeks	6.26	10 weeks
	*JBL*	12.1 eggs	16 weeks	100 %	8 weeks	2.8	8 weeks
	*Bloodworms*	39.8 eggs	20 weeks	59.5 %	7 weeks	8.3	11 weeks

Automatically Fed Aggregated average		**26.3 eggs**	**20 weeks**	**31.2 %**	**12 weeks**	**5.24**	**12 weeks**
Manually Fed Aggregated average		**19.7 eggs**	**18 weeks**	**55.2 %**	**8 weeks**	**4.18**	**8 weeks**

Color coding is used only to improve readability. Blue shading indicates male fish groups, pink shading indicates female fish groups, purple shading indicates automatically fed reproductive groups, and brown shading indicates manually fed reproductive groups. Grey shading is used for column grouping and does not indicate statistical significance or quantitative ranking.

## 4 Discussion

The present study introduces an innovative, custom-made automatic feeding system tailored to meet the specific housing requirements of the fish species *Nothobranchius furzeri*. This system offers a robust alternative to existing automated feeding technologies, which often suffer from high costs, complex infrastructure, or lack of precision (McKay et al., 2022; Lange et al., 2021; Doyle et al., 2017). Our design ensures a controlled and consistent supply of various dry foods over user-defined periods, eliminating the need for human intervention and reducing the risk of human errors. This innovation holds significant implications for experimental design and reliability, as it decouples feeding schedules from human working hours, enabling experiments during unconventional timeframes and ensuring mechanical synchronization for uniformity among experimental fish. Additionally, the system’s battery-free design and compatibility with standard electrical supply reduce maintenance and supervision time, while its modularity allows for easy removal and reconfiguration of feeding bars.

From a biological and husbandry perspective, the main value of the automated feeder is that it enables feeding frequency to be experimentally controlled at scale while reducing user-dependent variation in food delivery. This makes the system particularly useful for studies in which growth, lifespan, reproduction, or behavior may be affected by feeding timing, food availability, or diet composition.

Utilizing the automated feeding system, we administered four distinct types of food - Biomar, Otohime, JBL, and bloodworms - to different fish groups. The first three foods were chosen for their global commercial availability and distinct protein-to-fat ratios, as well as existing knowledge concerning their use with the Nothobranchius species (McKay et al., 2022; Žák et al., 2020). Biomar pellets exhibited the highest fat (18%) and protein (56%) content, surpassing that of bloodworms. Otohime, while sharing a similar protein content (50%) with Biomar and bloodworms, had lower fat content (10%). In contrast, JBL featured a minimal fat content (6%) but a significantly lower protein content (38%) compared to bloodworms. This selection allowed us to explore a broad spectrum of nutritional compositions, evaluating high, intermediate, and low-fat and protein foods and comparing them to bloodworms, providing insights into potential correlations with physiological parameters. Additionally, we employed the automatic feeding system to compare a continuous feeding protocol throughout the day with the standard twice-daily feeding regimen adopted by the majority of the researchers (Genade et al., 2005; Harel et al., 2015; Dodzian et al., 2018; Polačik et al., 2016). The automatic protocol fed the fish up to 36 times per day and was limited to the dry foods tested due to the system’s inability to dispense bloodworms, which nonetheless served as a reference diet. This automated feeding regimen represents the highest food delivery frequency tested to date, doubling the feeding intervals described in a previous study (McKay et al., 2022), and aimed to replicate the natural environment of Nothobranchius where food availability is constant throughout the day (Terzibasi Tozzini and Cellerino, 2020; Polačik and Reichard, 2010).

Fish growth, measured in both weight and length, was primarily influenced by feeding frequency rather than food type. Fish fed continuously throughout the day exhibited significantly higher weight and weight increase compared to those fed twice daily, despite receiving an equal total amount of food (Figure 5A). This discrepancy may indicate that continuous feeding increases effective food availability and/or nutrient uptake, potentially contributing to accelerated growth. It is important to note that the present study controlled the amount of food delivered to each group, but did not directly measure the amount of food ingested by individual fish. Therefore, feeding frequency and actual caloric intake cannot be fully separated. High-frequency automated feeding may have increased effective caloric intake by extending food availability throughout the day and reducing food loss, whereas manually fed fish may have experienced greater temporal variation in food access and/or uneaten food. Thus, the observed effects should be interpreted as consequences of the overall feeding regimen, including feeding frequency, delivered food amount, diet composition, and likely differences in actual intake. While food type had minimal impact on growth in continuously fed groups, manually fed groups showed a correlation between weight gain and the protein and fat content of the food. Biomar and Otohime induced weight gain rates comparable to bloodworms, consistent with previous findings (McKay et al., 2022; Žák et al., 2020), whereas JBL-fed fish displayed significantly delayed growth, likely due to its lower nutritional value. The lower content of fat and protein in JBL, or the absence of key nutrients, may account for this discrepancy. While effective in sustaining growth with continuous consumption, its efficacy diminishes when administered in a twice per day regimen, resulting in a suboptimal diet with adverse effects on fish development and growth. In terms of length increase, fish growth was generally similar between groups, except for manually fed JBL fish. Biomar and Otohime, when automatically fed, induced more substantial early-stage growth than manual or bloodworm feeding, though the difference was less pronounced than in weight. In later stages, the maximum length achieved eventually converged to similar values in all groups except for JBL manually fed fish. These trends were consistent across both male and female groups. Similar to what was observed for the weight, the outlier group was the JBL manually fed, leading to notably slow fish growth and reduced length. This anomaly may be again attributed to the very low fat and protein content or the absence of key nutrients, hindering proper nutrition for robust growth and resulting in underdeveloped fish. The observed discrepancy between fish growth and length suggests that these parameters are influenced by different factors, potentially including genetic factors (Dubois et al., 2012). While diet exhibited a linear effect on weight, with more calories correlating with greater weight, length demonstrated a threshold effect, where above a certain nutrient amount, fish developed comparably and caught up in later life stages, resulting in similar lengths.

Furthermore, our investigation into fish lifespan revealed unexpected results, indicating that the effects of food on longevity are independent of those on growth. Male fish exhibited similar median lifespans across all food types and feeding schedules. Automatic feeding groups experienced initial higher mortality, which later normalized, leading to similar median lifespans as other groups. This contrasts with previous research performed in this model (McKay et al., 2022), or in other vertebrate longevity model such as mice (Liao et al., 2010), where increased feeding rates or calorie supply is correlated with precocious male death. Despite the significantly larger weight of automatically fed fish, the observed comparable median longevity suggests that factors beyond diet, growth, or calories influence lifespan. The female group, however, portrayed a markedly distinct scenario, with fish fed automatically exhibiting a significantly shorter lifespan and a notable increase in premature deaths around week 7 but, unlike males, without subsequent recovery in median lifespan. This effect was not attributable to the food itself, as manually fed females receiving the same dry food lived significantly longer, even surpassing those fed bloodworms. We speculate that the reduced survival of automatically fed females may be linked to reproductive or ovary-related effects, as these females displayed increased body mass and an earlier loss of fertility. However, reproductive tissue morphology was not directly assessed in the present study, and this interpretation should therefore be considered only as a hypothesis for future investigation. These findings deviate from existing literature on killifish, where an *ad libitum* diet showed no impact on female lifespan (McKay et al., 2022), and in mice (Mitchell et al., 2016; Liao et al., 2010), where only or primarily male lifespan was affected by diet. Notably, our study involved higher and more frequent female feeding, as well as group housing, suggesting potential threshold effects or social dynamics influencing female survival. A limitation of the present study is that female fish were group-housed, whereas males were individually housed. Consequently, female survival and reproductive outcomes, as well as differences observed between males and females, may have been influenced not only by diet and feeding regimen, but also by tank-level and social-environmental factors. Although this housing design reflects the practical requirement for breeding and egg collection in N. furzeri, future studies with increased numbers of independent female tanks per treatment, and statistical models explicitly incorporating tank-level effects, will be necessary to formally quantify these effects and further exclude pseudoreplication.

Collectively, the pooled reproductive profiles suggest that reproductive output varied with both diet composition and feeding regimen. Because embryos were pooled by treatment group and tank identity was not retained, these reproductive data should be interpreted descriptively rather than as statistically robust treatment effects. Within this limitation, higher-fat and higher-protein diets were associated with higher pooled egg production but lower pooled embryo survival and a shorter reproductive window. These patterns appeared more pronounced under automatic feeding, whereas manually fed groups showed lower but more prolonged reproductive output.

Bloodworm-fed groups showed the most sustained pooled reproductive profile, with high egg production and embryo survival across much of the reproductive period.

JBL, when automatically fed, showed a relatively favorable pooled reproductive profile, with lower egg production but comparatively high embryo survival. This suggests that, under high-frequency feeding conditions, this lower-fat and lower-protein dry diet may still provide sufficient nutritional support for embryo viability. In contrast, manual feeding of JBL was associated with limited egg production over an extended period, resulting in a low overall reproductive score despite relatively high embryo survival.

As previously noted for other physiological parameters, this pattern may reflect the lower nutritional value of JBL, which may be sufficient only when food availability is distributed across the day. However, these interpretations should be considered hypothesis-generating and will require future experiments preserving tank-level replication to test statistically.

In conclusion, our findings suggest that, under controlled laboratory conditions, the relationship between delivered food availability, diet composition, and reproductive output in laboratory N. furzeri may be more complex than predicted by a simple resource-availability model (Endara and Coley, 2011). This model is based on the premise that increased resource availability can support reproduction because individuals have access to more energy and nutrients (Hagmayer et al., 2022; Holliday, 1989). However, our data suggest that greater delivered food availability and energy-rich diets may promote egg production in the short term, but do not necessarily support higher embryo survival or sustained reproductive performance over time. Instead, our results indicate that diet composition and feeding regimen may need to be balanced according to the specific experimental goal, particularly when both fecundity and embryo viability are relevant outcomes. Together, these observations suggest that, under laboratory husbandry conditions, diet composition and quality may be more important than food abundance alone for sustaining reproductive performance, potentially aligning with observations in other species in which excessive food availability is associated with increased body mass and reproductive impairment (Magwere et al., 2004; Dağ and Dilbaz, 2015; Costa et al., 2019; Adatto et al., 2022). However, this interpretation should not be directly extrapolated to natural populations without further ecological validation.

## Data Availability

The original contributions presented in the study are included in the article/Supplementary Material, further inquiries can be directed to the corresponding authors.

## Supplementary material

SUPPLEMENTARY FIGURE S1 Automatic Feeder Building Instructions. Step-by-step visual guide for construction and wiring of the automated feeding system. **(A)** Mechanical assembly of the feeder, including cutting of the aluminum bar, preparation and positioning of plastic tube holders, printing and insertion of the 3D motor-bar adaptors, ball-bearing holder preparation, motor-side assembly, and fixation of the assembled feeder to the aquarium scaffold. **(B)** Electrical assembly of the feeder, including connection of the stepper motor to the ULN2003 driver board, Arduino programming with Supplementary Material feeding.ino, preparation of connection wires, wiring of the ULN2003 board to the Arduino and external 5 V power supply, connection of the Arduino to a digital timer synchronized with the room light phase, and optional parallel wiring for simultaneous control of multiple feeder bars.

SUPPLEMENTARY TABLE S1 Complete statistical results for growth analyses related to Figure 5. The table reports pairwise comparisons for body weight and body length, including significance levels, exact p-values, and quantitative effect estimates. Effect estimates were calculated using raw body weight and length measurements at 18 weeks of age, expressed as the absolute mean difference between the two compared groups (Δ mean; first group listed minus second group listed) and as Hedges’ g, a standardized mean difference corrected for small sample size. The 18-week time point was selected because it represents an adult stage at which growth differences were well established while sufficient measurements were still available across experimental groups before later survival-related reductions in sample size. Positive effect estimate values indicate higher body weight or length in the first group listed in the comparison.

SUPPLEMENTARY TABLE S2 Complete statistical results for survival analyses related to Figure 6. The table reports pairwise comparisons of Kaplan–Meier survival curves, including significance levels, exact p-values from log-rank (Mantel–Cox) tests, and quantitative effect estimates. Effect estimates are expressed as differences in median survival between the compared groups (Δ median survival; first group listed minus second group listed, in weeks). Positive values indicate longer median survival in the first group listed in the comparison, whereas negative values indicate shorter median survival in the first group listed.

SUPPLEMENTARY TABLE S3 Feeding regimen across experimental age phases. The table summarizes the automatic and manual feeding schedules used from 7 days post-hatching onward, including feeding frequency, rotations per feeding event, estimated dry-food amount delivered per fish per day, and tube-opening adjustments used to tune dry-food delivery for different foods and housing conditions. Manual dry-food groups received the same total daily food amount as the corresponding automatic dry-food groups, divided into two feedings per day.

SUPPLEMENTARY VIDEO S1 Operation of an installed Automatic Feeding System. The video shows two full rotations of a feeding event for individually housed fish. Tubes are loaded with distinct dry foods corresponding to each fish’s diet.

SUPPLEMENTARY VIDEO S2 Close-up of Food Dispensation. The video provides a close-up view of a feeding event, showcasing the precise dropping of dry food into the fish tank **(A)**. Food pellets are visible as they are dispensed onto the water surface **(B)**.

SUPPLEMENTARY VIDEO S3 Motor and Control Board functioning Close-up. The video illustrates the motor control system during feeding. LEDs on the ULN-2003 board flash and sequentially light up **(A)**, indicating that the automatic feeding is about to commence. Subsequently, the motor rotates, driving the entire feeding bar rotation **(B)**.
