# Supplementary material for: Sex-specific effects of automated feeding on lifespan and reproduction in Nothobranchius furzeri
Source: Front Aging. 2026 Sep 14;7:1869084. doi: 10.3389/fragi.2026.1869084 (PMC13616631; doi:10.3389/fragi.2026.1869084)
Supplement: Supplementary file 1 [file DataSheet1.pdf]

Supplementary Technical Protocol

Automated Feeding System Construction, Programming, and Food Delivery Validation

1. Overview

This Supplementary Technical Protocol provides detailed information for reproducing the automated feeding system described in the main manuscript. The system was designed for high-frequency dry-food delivery in standard *Nothobranchius furzeri* aquaria. It consists of a motor-driven feeding bar positioned above a row of tanks, an Arduino-controlled rotation system and removable dry-food tubes aligned with individual aquaria, with openings that allow gravity-driven food release during bar rotation.

The protocol is divided into four main parts: feeder construction, electrical wiring and rotation control, aquarium installation and lid modification, and food delivery validation. Component specifications and supplier information are summarized in the final component table. Equivalent components can be used if they match the same functional specifications.

---

2. Automated Feeder Construction (Figure 1A,B)

2.1. Feeding bar

The main feeding unit was built using an L-shaped aluminum bar (1h). The bar was cut to a length corresponding to the distance between the aquarium scaffold bars minus 7 cm. This allowed the assembled feeding bar to fit between the lateral scaffold supports while leaving sufficient space for the terminal holders and motor connection.

At both ends of the aluminum bar, 3 cm long round plastic tubes (1j) with a diameter of 2 cm were attached using silicone. These terminal tubes served as connection points between the aluminum bar and the 3D-printed motor-bar adaptors (1f).

2.2. Food tube holders

Food-tube holders (1k) were prepared from 2 cm long plastic tube segments. Each segment was partially opened by cutting away approximately 25% of the tube wall, creating a semi-open clip able to hold a removable dry-food tube. These holders were attached to the aluminum bar using silicone.

The spacing of the holders was adjusted according to the spacing of the tanks in the aquarium row, so that each removable food tube (1o) was positioned directly above the corresponding tank. This alignment allowed food to be released into the correct tank during bar rotation.

2.3. Frame holders and ball bearings

To mount the feeding bar on the aquarium scaffold (1q), square frame holders (1m,n) were handcrafted from PVC-foam plates. Each holder measured approximately 5 × 5 cm and contained four 4 mm openings near the edges for fixation screws (1l). A larger central opening of approximately 2.2 cm was made to accommodate a ball bearing (1i).

Ball bearings were inserted into the central opening of the frame holders. The ball bearings supported the rotating axis of the feeding bar and reduced friction during motor-driven rotation.

Two types of frame holders were used: one standard holder (1m) and one motor-side holder (1n). The motor-side holder supported the stepper motor and allowed the motor rotor to align with the rotating feeding bar.

2.4. Stepper motor and 3D-printed adaptors

A 28BYJ-48 stepper motor (1g) was mounted on the motor-side frame holder. The motor rotor was aligned with the central ball-bearing opening and fixed in place using two 8 mm wood screws.

Two 3D-printed motor-bar adaptors (1f) were generated from the supplementary file adaptor.stl. The adaptors were printed in PLA. For sufficient mechanical strength, an infill above 35% is recommended.

Each adaptor contains a smaller cylindrical part and a larger cylindrical part. The smaller cylinder was inserted into the ball-bearing (1i) opening and connected to the motor rotor, while the larger cylinder was inserted into the terminal plastic tube at the end of the aluminum bar. This configuration mechanically connected the motor to the feeding bar and allowed stable rotation of the full bar.

---

3. Aquarium Installation and Lid Modification (Figure 1C,D)

The assembled feeding bar was installed approximately 8 cm above the tanks. The frame holders were positioned around the aquarium scaffold bars and secured using 6 cm screws and bolts (1l). Once installed, the feeding bar was able to rotate above the row of tanks while keeping each food tube aligned with the corresponding aquarium (Video S1).

Tank lids were modified to allow food entry during feeder rotation. An approximately 8 cm wide opening was created in each lid below the position of the corresponding food tube. The opening was covered with a grid to prevent fish escape while allowing food to fall into the tank. The grid-covered opening ensured that the food released during bar rotation was deposited inside the correct aquarium (Video S2).

---

4. Electrical Wiring and Motor Control (Figure 1E)

Each 28BYJ-48 stepper motor was connected to a ULN2003 driver board (1e). The ULN2003 board was fixed on the outer side of the scaffold adapter and connected to the motor using the designated motor cable.

From each ULN2003 board, six cables (1c) were used for Arduino UNO (1b) and power-supply connection: 5V, GND, IN1, IN2, IN3, and IN4. Cable ends were fitted with Dupont connectors (1d) to allow stable insertion into the Arduino and power-supply connectors.

The 5V and GND lines were connected to a 1000 mA 5V power supply (1a). The control lines were connected to the Arduino using the following configuration:

ULN2003 output	Arduino pin
IN1	pin 11
IN2	pin 10
IN3	pin 9
IN4	pin 8

Multiple ULN2003 boards could be connected to the same Arduino to control several motor-bar units simultaneously. In this configuration, the corresponding IN1, IN2, IN3, and IN4 cable lines from multiple driver boards were connected in parallel wiring the cables at the junctions and then directed to the corresponding Arduino pin as a single control line. This allowed synchronized rotation of multiple feeding bars (Figure 1E).

Because all motors connected to the same Arduino received the same commands, individual motor programming was not possible in this configuration. Food delivery to individual tanks was therefore adjusted mechanically by changing the diameter and/or number of openings drilled in the removable food tubes (1o).

---

5. Arduino Feeding Program

The Arduino was connected to a computer via USB and programmed using the Arduino IDE software. The feeding script is provided as Supplementary File feeding.ino.

The program was designed to perform two types of movements:

1. **Full rotations for food delivery**
   During each feeding event, the motor performed two full rotations, one clockwise and one anticlockwise. In the program used here, one full rotation corresponded to 2000 motor steps. During each feeding event, the stepper motor rotated the entire aluminum bar together with the attached food tubes. These full rotations allowed dry food to fall through the tube openings into the tanks below.
2. **Partial rotations for tube shaking**
   Between feeding events, the motor performed short alternating partial rotations (shaking) to keep the dry food mobile inside the tubes and reduce aggregation. In the program used here, shaking consisted of four 600-step movements, alternating

clockwise and anticlockwise. These shaking movements were performed every 5 minutes.

In the highest-frequency feeding program, feeding events occurred every 20 minutes. During the 12-hour light phase, this corresponds to 36 feeding events per day. Lower feeding frequencies, including 12 and 24 feeding events per day, were obtained by increasing the interval between full-rotation feeding events.

Once the program was uploaded to the Arduino, the script started from the beginning each time the Arduino was powered on and then operated in a continuous loop.

Each Arduino was connected to a digital timer synchronized with the room 12-hour light/12-hour dark photoperiod. The timer switched the Arduino on during the light phase and off during the dark phase. This ensured that the feeding program started with the onset of light and that food delivery occurred only during daytime, preventing nocturnal food delivery.

---

6. Food Tube Preparation and Adjustment of Food Delivery

Removable food tubes (1o) were filled with dry food and inserted into the tube holders (1k) on the feeding bar. Before use, circular openings were manually drilled in the middle region of each tube using drill bits of defined diameter. These drilled openings controlled the amount of food released during rotation (Figure 1C). Different tube-opening diameters were used to tune food delivery according to food type, fish age, housing condition, and required daily food amount.

For food delivery testing, three opening diameters were used: 1.5 mm, 2.0 mm, and 2.5 mm. Larger openings released more food per feeding event, while smaller openings provided lower and more precise food delivery. Because dry foods differ in granule size, shape, and density, food delivery was calibrated separately for each diet.

In the experimental setup, food tubes were filled with 10 g of dry food and refilled weekly to restore the amount to 10 g. During use, the 5-minute shaking intervals helped prevent food aggregation inside the tubes.

---

7. Food Delivery Testing Setup (Figure 3A,B)

Food delivery precision was quantified using a reduced feeder setup coupled to an Arduino-compatible load-cell scale (3g). The setup was designed to measure the amount of dry food released during repeated feeding events.

The testing frame was built from 2 cm thick wood. The frame measured approximately 27 cm in height, 30 cm in width, and 20 cm in depth. Two 2 cm openings were drilled into the main wooden frame to accommodate ball bearings. A small version of the L-shaped aluminum feeding bar (3d) was inserted between the ball bearings and connected using two 3D-printed adaptors.

Only one food-tube holder was positioned in the middle of the reduced aluminum bar, allowing one food tube to be tested at a time. A 28BYJ-48 stepper motor (3c), ULN2003 driver board (3b), and Arduino (3a) were mounted on one side of the wooden frame. The stepper motor was connected to the reduced feeding bar through the 3D-printed adaptor.

The motor was connected to the ULN2003 board, which was connected to the Arduino using the same configuration used for the full feeder:

ULN2003 output	Arduino pin
5V	5V
GND	GND
IN1	pin 11
IN2	pin 10
IN3	pin 9
IN4	pin 8

A block of Styrofoam was fixed to the wooden plate of the frame to reduce vibration transfer to the high-precision digital load-cell scale (3g). The scale was mounted using a 5 × 8 cm, 3 mm thick aluminum sheet as weighing plate and a 5 × 13 cm, 3 mm thick aluminum sheet as base.

The load-cell scale was connected to an HX711 Load Cell Weight Sensor Module (3h) using the E+, E-, A-, and A+ pins. The HX711 module was then connected to the Arduino using the following configuration:

HX711 module	Arduino pin
VCC	3.3V
GND	GND
DT	pin 3
SCK	pin 4

A collection vessel (3f) was placed on the scale weighing plate below the food tube to collect food released during each feeding event.

---

8. Food Delivery Validation

Food delivery was tested using three dry foods: BioMar, Otohime, and JBL. Three independent food tubes, labeled A, B, and C, were used as technical replicates. Each tube contained three possible opening diameters: 1.5 mm, 2.0 mm, and 2.5 mm.

For each test, only one opening was left exposed, while the other openings were sealed with tape. Each food type was tested in each tube and for each opening diameter, resulting in three technical replicates per food/opening combination and 27 total tests.

Before each test, the food tube was loaded with exactly 10 g of dry food and inserted into the tube holder on the reduced feeder. The bar was manually positioned so that the tube opening faced upward before starting the test. An empty collection vessel was placed on the weighing plate to collect the dispensed food.

For each experiment, the Arduino was connected to a computer (3i) via USB, and the Arduino IDE software was used to run the testing script. The serial monitor was opened with a baud rate of 115200 to record the digital signal from the load-cell scale.

The testing program performed two full rotations per feeding event, one clockwise and one anticlockwise, allowing food to be released onto the scale twice. After a 5-second pause, the weight signal was recorded 20 times over 2 seconds and averaged to obtain a more stable output. The program then repeated this loop until manually stopped. Each experiment continued for at least 1 hour and included more than 400 repeated measurements (Figure 3C).

---

9. Calibration and Calculation of Food Delivery

The load-cell scale output was recorded as a digital signal and later converted to grams. For each test, the first recorded value was used as the zero-reference signal. At the end of the experiment, the total amount of food dispensed was measured using an independent scale. This final measured mass was used to calculate the conversion ratio between the digital scale signal and grams.

For the analysis of food delivery, 24 feeding events were used as the standard measurement block. This number was selected because it corresponds to one of the experimental feeding schedules used in the study: 24 feeding events during the 12-hour light phase, equivalent to two feeding events per hour. Using this fixed block size allowed food delivery to be compared across food types and tube-opening diameters under a standardized daily feeding routine.

For each food/opening combination, food delivery was calculated from 10 consecutive blocks of 24 feeding events. This corresponded to a total of 240 feeding events per food/opening combination. Because each feeding event consisted of two full rotations, one clockwise and one anticlockwise, each feeding event generated two individual food drops. Therefore, 240 feeding events corresponded to 480 individual food drops.

For each 24-event block, the amount of food delivered per feeding event was calculated. The average of the 10 blocks was then used to determine the mean food delivery for each food type and tube-opening diameter. The 10 repeated blocks were also compared with each other to assess whether food delivery remained stable over time during repeated feeder operation (Figure 3D).

---

10. Practical Notes and Troubleshooting

- **Food delivery depends on food physical properties.** Granule size, shape, density, and surface texture influence the amount of food released during each rotation.

- **Opening diameter strongly affects delivery.** Larger openings release more food but may reduce fine control, whereas smaller openings allow lower and more precise delivery.
- **Food aggregation should be minimized.** Partial shaking rotations every 5 minutes help prevent food clumping inside the tubes.
- **Moisture should be monitored.** Food tubes should be checked regularly for moisture accumulation, mold, or obstruction.
- **All motors connected to one Arduino rotate synchronously.** Individual feeding amounts are adjusted mechanically through tube-opening size and/or number, not by independent motor programming.
- **Tube alignment is important.** Food tubes should be aligned with the corresponding tank opening to ensure complete food deposition inside the aquarium.
- **Testing is recommended after tube modification.** Any change in food type, opening diameter, or number of openings should be validated before use in long-term experiments.

---

11. Component Table

Figure label	Component	Supplier/source used	Catalog/product number	Function/notes
1a	5V power supply adapter	Reichelt	GOOBAY 67951	1000 mA, 5V power supply for motor-driver system
1b/3a	Arduino UNO	Reichelt	ARDUINO UNO	Microcontroller used to control feeder rotation
1c	Cable connectors	Reichelt	LITZE RT/SW/GN/GE/BR/GR	Connection cables for power and control lines
1d	Dupont pin connectors	Reichelt	DEBO SET DUPONT	Used for stable cable insertion into Arduino/driver boards
1e/3b	ULN2003 driver board	Reichelt	DEBO DRV2ULN2003	Driver board for 28BYJ-48 stepper motors
1f	3D-printed motor-bar connectors	Custom printed	Supplementary File adaptor.stl	Connects motor rotor and feeding bar through ball-bearing support
1g/3c	28BYJ-48 stepper motor	Reichelt	28BYJ-48	Motor used to rotate the feeding bar
1h/3d	L-shaped aluminum bar	Hornbach	Art-Nr. 8829144	Main rotating feeding bar
1i	Ball bearings	Hornbach	Art-Nr. 6644713	Support rotation of the feeding bar
1j	Terminal plastic tube	Hornbach	Art-Nr. 5715556	Plastic tube segments attached to both ends of

Figure label	Component	Supplier/source used	Catalog/product number	Function/notes
	connectors			the aluminum bar
1k	Food-tube holders	Hornbach/custom prepared	Art-Nr. 5715556	Semi-open plastic clips used to hold removable food tubes
1l	Screws, bolts, and nuts	Hornbach	Art.-Nr. 6836246	Used to secure frame holders to aquarium scaffold
1m	Standard frame holder	Hornbach/custom prepared	Art-Nr. 3887999	Handmade PVC-foam holder used to mount feeder on scaffold
1n	Motor-side frame holder	Hornbach/custom prepared	Art-Nr. 3887999	Handmade PVC-foam holder supporting the motor and rotating bar
1o/3e	Removable food tubes	djb labcare	Art-Nr. 309202	Tubes containing dry food
—	Digital timer	Hornbach	Art-Nr. 6376083	Switches Arduino on/off according to the 12-hour light/12-hour dark cycle
—	Load-cell scale	Amazon	https://www.amazon.de/-/en/Module-Weight-Weighing-Pressure-Measurement/dp/B0GL21VR1G/ref=sr_1_3?sr=8-3	High-precision (0-1Kg) digital scale for food delivery testing
—	HX711 Load Cell Weight Sensor Module	Amazon	https://www.amazon.de/-/en/Module-Weight-Weighing-Pressure-Measurement/dp/B0GL21VR1G/ref=sr_1_3?sr=8-3	Converts load-cell signal for Arduino acquisition
