# Supplementary material for: Sex-specific effects of automated feeding on lifespan and reproduction in Nothobranchius furzeri
Source: Front Aging. 2026 Sep 14;7:1869084. doi: 10.3389/fragi.2026.1869084 (PMC13616631; doi:10.3389/fragi.2026.1869084)
Supplement: Supplementary file 2 [file DataSheet2.pdf]

Age phase	Fish age	Automatic feeding frequency	Rotations per feeding event	Dry food delivered per fish per day	Manual dry-food feeding	Tube openings used for automatic feeding
I	7–21 days post-hatching	24 feedings/day	2 rotations	~18 mg/fish/day	Twice daily; each feeding delivered half of the corresponding automatic daily amount	2 mm for BioMar and JBL; two 2 mm openings for Otohime
II	21–42 days post-hatching	12 feedings/day	2 rotations	~30 mg/fish/day	Twice daily; each feeding delivered half of the corresponding automatic daily amount	1.5 mm for single-housed males; 2 mm for group-housed females; two openings for Otohime
III	42–70 days post-hatching	24 feedings/day	2 rotations	~55 mg/fish/day	Twice daily; each feeding delivered half of the corresponding automatic daily amount	1.5 mm for single-housed males; 2 mm for group-housed females; two openings for Otohime
IV	70 days post-hatching onward	36 feedings/day	2 rotations	~80 mg/fish/day	Twice daily; each feeding delivered half of the corresponding automatic daily amount	1.5 mm for single-housed males; 2 mm for group-housed females; two openings for Otohime
