# Supplementary material for: Sex-specific effects of automated feeding on lifespan and reproduction in Nothobranchius furzeri
Source: Front Aging. 2026 Sep 14;7:1869084. doi: 10.3389/fragi.2026.1869084 (PMC13616631; doi:10.3389/fragi.2026.1869084)
Supplement: Supplementary file 3 [file DataSheet3.pdf]

male weight				
**comparison**	**significance**	**exact p-value**	**Δ mean at 18 weeks (g)**	**Hedges' g at 18 weeks**
**aggregated**				
bloodworms vs auto	***	*2.6206E-38*	*-0.893*	*-1.90*
bloodworms vs manual	***	8.5789E-08	0.378	0.66
auto vs manual	***	1.9818E-67	1.271	1.84
**single auto**				
bloodworms vs biomar	***	*7.7443E-33*	*-1.043*	*-3.58*
bloodworms vs otohime	***	3.3434E-33	-0.966	-2.31
bloodworms vs JBL	***	5.6357E-19	-0.595	-1.49
biomar vs otohime	ns	0.7293	0.077	0.14
biomar vs JBL	*	0.0131	0.448	0.83
otohime vs JBL	*	0.0188	0.372	0.54
**single manual**				
bloodworms vs biomar	*ns*	*0.0634*	*-0.206*	*-0.81*
bloodworms vs otohime	ns	0.5324	-0.126	-0.47
bloodworms vs JBL	***	4.7662E-56	1.320	4.56
biomar vs otohime	ns	0.0634	0.080	0.24
biomar vs JBL	***	8.4489E-52	1.527	4.05
otohime vs JBL	***	3.4534E-37	1.447	3.71

female weight				
**comparison**	**significance**	**exact p-value**	**Δ mean at 18 weeks (g)**	**Hedges' g at 18 weeks**
**aggregated**				
bloodworms vs auto	***	*1.2871E-14*	*-0.536*	*-1.05*
bloodworms vs manual	*	0.0201	0.052	0.12
auto vs manual	***	4.3553E-24	0.588	1.05
**single auto**				
bloodworms vs biomar	***	*2.0068E-24*	*-0.843*	*-2.57*
bloodworms vs otohime	***	1.6478E-24	-1.037	-4.65
bloodworms vs JBL	ns	0.7897	0.080	0.36
biomar vs otohime	ns	0.8027	-0.194	-0.45
biomar vs JBL	***	6.5008E-21	0.923	2.30
otohime vs JBL	***	6.5008E-21	1.116	3.97
**single manual**				
bloodworms vs biomar	***	*5.5289E-06*	*-0.314*	*-1.04*
bloodworms vs otohime	ns	0.2135	0.002	0.01
bloodworms vs JBL	***	2.4499E-25	0.629	2.38
biomar vs otohime	***	1.2248E-06	0.316	0.95
biomar vs JBL	***	1.4511E-32	0.943	2.32
otohime vs JBL	***	2.2300E-17	0.627	2.09

male length				
**comparison**	**significance**	**exact p-value**	**Δ mean at 18 weeks (cm)**	**Hedges' g at 18 weeks**
**aggregated**				
bloodworms vs auto	***	*5.9339E-08*	*-0.091*	*-0.21*
bloodworms vs manual	***	1.1543E-18	0.736	1.00
auto vs manual	***	1.5384E-44	0.827	1.09
**single auto**				
bloodworms vs biomar	***	*5.2839E-12*	*-0.312*	*-0.95*
bloodworms vs otohime	***	1.5934E-17	-0.262	-0.82
bloodworms vs JBL	ns	0.3337	0.432	1.18
biomar vs otohime	ns	0.3337	0.050	0.14
biomar vs JBL	***	4.1690E-06	0.743	1.72
otohime vs JBL	***	3.3994E-09	0.693	1.65
**single manual**				
bloodworms vs biomar	*ns*	*0.8645*	*0.119*	*0.39*
bloodworms vs otohime	ns	0.5794	-0.026	-0.09
bloodworms vs JBL	***	2.7434E-72	1.960	4.94
biomar vs otohime	ns	0.8645	-0.146	-0.62
biomar vs JBL	***	7.5362E-54	1.841	3.56
otohime vs JBL	***	7.3905E-48	1.987	4.19

female length				
**comparison**	**significance**	**exact p-value**	**Δ mean at 18 weeks (cm)**	**Hedges' g at 18 weeks**
**aggregated**				
bloodworms vs auto	*	*0.0135*	*-0.034*	*-0.07*
bloodworms vs manual	***	4.3933E-07	0.525	0.94
auto vs manual	***	1.8887E-15	0.560	0.94
**single auto**				
bloodworms vs biomar	***	*1.1257E-06*	*0.028*	*0.07*
bloodworms vs otohime	***	1.1257E-06	-0.546	-2.08
bloodworms vs JBL	**	0.0046	0.331	1.05
biomar vs otohime	ns	0.8925	-0.574	-1.31
biomar vs JBL	***	1.4208E-11	0.304	0.64
otohime vs JBL	***	1.1418E-11	0.878	3.10
**single manual**				
bloodworms vs biomar	*ns*	*0.6772*	*0.164*	*0.47*
bloodworms vs otohime	ns	0.6772	0.302	1.07
bloodworms vs JBL	***	1.2715E-39	1.356	3.32
biomar vs otohime	ns	0.6490	0.139	0.40
biomar vs JBL	***	3.1923E-32	1.192	2.43
otohime vs JBL	***	5.2372E-30	1.054	2.54
