# Supplementary material for: Sex-specific effects of automated feeding on lifespan and reproduction in Nothobranchius furzeri
Source: Front Aging. 2026 Sep 14;7:1869084. doi: 10.3389/fragi.2026.1869084 (PMC13616631; doi:10.3389/fragi.2026.1869084)
Supplement: Supplementary file 4 [file DataSheet4.pdf]

| male | | | |
|---|---|---|---|
| comparison | signif. * | p-value | effect estimate (Delta median survival, weeks) |
| **aggregated** | | | |
| bloodworms vs auto | ns | 0.1107 | -0.7 |
| bloodworms vs manual | ns | 0.9316 | 1.3 |
| auto vs manual | ns | 0.1417 | 2.0 |
| | | | |
| **single auto** | | | |
| bloodworms vs biomar | ns | 0.3559 | -0.7 |
| bloodworms vs otohime | ns | 0.4133 | -1.5 |
| bloodworms vs JBL | ns | 0.1844 | 1.7 |
| biomar vs otohime | ns | 0.8369 | -0.8 |
| biomar vs JBL | ns | 0.9211 | 2.4 |
| otohime vs JBL | ns | 0.6296 | 3.2 |
| | | | |
| **single manual** | | | |
| bloodworms vs biomar | ns | 0.2431 | 3.3 |
| bloodworms vs otohime | ns | 0.4352 | 0.4 |
| bloodworms vs JBL | ns | 0.3126 | -3.1 |
| biomar vs otohime | ns | 0.8899 | -2.9 |
| biomar vs JBL | ns | 0.1192 | -6.4 |
| otohime vs JBL | ns | 0.1688 | -3.5 |

| female | | | |
|---|---|---|---|
| comparison | signif. * | p-value | effect estimate (Delta median survival, weeks) |
| **aggregated** | | | |
| bloodworms vs auto | * | 0.0464 | 4.0 |
| bloodworms vs manual | ** | 0.0050 | -2.8 |
| auto vs manual | **** | < 0.0001 | -6.8 |
| | | | |
| **single auto** | | | |
| bloodworms vs biomar | ** | 0.0037 | 4.0 |
| bloodworms vs otohime | * | 0.0157 | 6.6 |
| bloodworms vs JBL | ns | 0.9215 | 1.5 |
| biomar vs otohime | ns | 0.7526 | 2.6 |
| biomar vs JBL | * | 0.0440 | -2.5 |
| otohime vs JBL | * | 0.0276 | -5.1 |
| | | | |
| **single manual** | | | |
| bloodworms vs biomar | ns | 0.1307 | -2.6 |
| bloodworms vs otohime | * | 0.0441 | -2.8 |
| bloodworms vs JBL | * | 0.0182 | -2.9 |
| biomar vs otohime | ns | 0.5406 | -0.2 |
| biomar vs JBL | ns | 0.2310 | -0.3 |
| otohime vs JBL | ns | 0.2180 | -0.1 |
